# Co-delivery of VEGF siRNA and Etoposide for Enhanced Anti-angiogenesis and Anti-proliferation Effect *via* Multi-functional Nanoparticles for Orthotopic Non-Small Cell Lung Cancer Treatment

**DOI:** 10.7150/thno.32416

**Published:** 2019-08-12

**Authors:** Fang Li, Yu Wang, Wei-liang Chen, Dan-dan Wang, Ye-juan Zhou, Ben-gang You, Yang Liu, Chen-xi Qu, Shu-di Yang, Meng-tian Chen, Xue-nong Zhang

**Affiliations:** 1 Department of Pharmaceutics, College of Pharmaceutical Sciences, Soochow University, Suzhou 215123, People's Republic of China; 2 Department of Pharmacy, Children` s Hospital of Soochow University, Suzhou 215025, People's Republic of China

**Keywords:** anti-angiogenesis, combined therapy, co-delivery, multi-functional nanoparticles, VEGF siRNA, etoposide

## Abstract

Targeting tumor angiogenesis pathway *via* VEGF siRNA (siVEGF) has shown great potential in treating highly malignant and metastatic non-small cell lung cancer (NSCLC). However, anti-angiogenic monotherapy lacked sufficient antitumor efficacy which suffered from malignant tumor proliferation. Therefore, the combined application of siVEGF and chemotherapeutic agents for simultaneous targeting of tumor proliferation and angiogenesis has been a research hotspot to explore a promising NSCLC therapy regimen.

**Methods:** We designed, for the first time, a rational therapy strategy *via* intelligently co-delivering siVEGF and chemotherapeutics etoposide (ETO) by multi-functional nanoparticles (NPs) directed against the orthotopic NSCLC. These NPs consisted of cationic liposomes loaded with siVEGF and ETO and then coated with versatile polymer PEGylated histidine-grafted chitosan-lipoic acid (PHCL). We then comprehensively evaluated the anti-angiogenic and anti-proliferation efficiency in the *in vitro* tumor cell model and in bioluminescent orthotopic lung tumor bearing mice model.

**Results:** The NPs co-delivering siVEGF and ETO exhibited tailor-made surface charge reversal features in mimicking tumor extracellular environment with improved internal tumor penetration capacity and higher cellular internalization. Furthermore, these NPs with flexible particles size triggered by intracellular acidic environment and redox environment showed pinpointed and sharp intracellular cargo release guaranteeing adequate active drug concentration in tumor cells. Enhanced VEGF gene expression silencing efficacy and improved tumor cell anti-proliferation effect were demonstrated *in vitro*. In addition, the PHCL layer improved the stability of these NPs in neutral environment allowing enhanced orthotopic lung tumor targeting efficiency *in vivo*. The combined therapy by siVEGF and ETO co-delivered NPs for orthotopic NSCLC simultaneously inhibited tumor proliferation and tumor angiogenesis resulting in more significant suppression of tumor growth and metastasis than monotherapy.

**Conclusion:** Combined application of siVEGF and ETO by the multi-functional NPs with excellent and on-demand properties exhibited the desired antitumor effect on the orthotopic lung tumor. Our work has significant potential in promoting combined anti-angiogenesis therapy and chemotherapy regimen for clinical NSCLC treatment.

## Introduction

Non-small cell lung cancer (NSCLC) has been reported to be multifactorial disease with high mortality and morbidity 1. Abundant blood vessels in lung tumor sites not only provide nutrients and oxygen for malignant proliferation and progression but also act as vehicles for disseminating the primary tumor to other sites to form secondary foci and subsequently result in uncontrolled tumor metastasis 2-4. Targeted tumor angiogenesis therapy through anti-angiogenic agents has been explored for advanced NSCLC treatment 5. However, due to the malignant proliferation of tumor cells, anti-angiogenic monotherapy has been proven to have insufficient and short-term anticancer efficacy despite temporary tumor suppression in the early treatment stage of NSCLC 6. Chemotherapy alone based on chemotherapeutics has shown high efficacy in inhibiting tumor proliferation during the early treatment of NSCLC but was ineffective against uncontrolled tumor angiogenesis and tumor metastasis 7, 8. To address the dilemma, integrating the potency of chemotherapy and anti-angiogenic therapy would benefit from the combined inhibition of tumor cell apoptosis and tumor angiogenesis and lead to long-term suppression of tumor growth and metastasis for advanced NSCLC 9.

Vascular endothelial growth factor (VEGF) is overexpressed in most malignant tumors and significantly promotes tumor neovascularization 10-12. Targeted angiogenesis therapy through blocking the VEGF signaling pathway has shown considerable promise in advanced NSCLC treatment 13. VEGF small interference RNA (siRNA) can precisely and efficiently silence VEGF expression and block VEGF signal pathway, leading to a significant decrease in tumor blood vessels and suppression of tumor growth and metastasis 14-16. However, siRNA with an inherent negative charge, high hydrophilicity and macromolecular size, is susceptible to ribonuclease (RNase) degradation and suffers from poor stability and low targeted tumor accumulation during the delivery process 17. Etoposide (ETO) with poor solubility, a topoisomerase II (topo II) inhibitor that induces tumor cell apoptosis has been reported to effective chemotherapeutic agent for NSCLC. Unfortunately, non-selective accumulation of ETO *in vivo* causes severe side effects to normal tissues 18. Therefore, it is of great urgency to rationally design the co-delivery system to incorporate ETO agent and siRNA with high efficiency and safety *in vivo*.

Nano-vehicles have been extensively developed for effective delivery of siRNA or hydrophobic chemotherapeutics 19. Positively charged cationic liposomes (Lip) have been widely used as siRNA and chemotherapeutic carriers because of high loading capacity and significantly enhanced cellular internalization through adsorptive interactions with tumor cell membrane 20. Unfortunately, positively charged cationic Lip suffer from aggregation and easy clearance from blood circulation due to the non- specific electrostatic adsorption with blood components and quick recognition by the immune system 21. Despite PEGylation used to shield the positive charge of nanoparticles (NPs) for improved circulation, these NPs still exhibit low positive charge, causing insufficient stability in blood circulation 22, 23. Negatively charged NPs formed from anionic polymer have been proved to highly resist non- specific electrostatic adsorption in blood circulation resulting in prolonged circulation and enhanced accumulation in tumor sites by enhanced permeability and retention (EPR) effect 24. Anionic polymers are promising alternatives for positively charged NPs coating materials 25. Unfortunately, Schlegel et al. designed anionic polymer-modified cationic Lip to shield the strong positive charge but the negative charge of NPs hindered cell internalization 26. Hence, it is of great importance to develop smart NPs with tailor-made surface charge property to simultaneously avoid non-specific interaction for long circulation and enhance cell internalization after accumulation in tumor sites. Stimuli-sensitive polymer with charge-switchable property that exhibit negative charge in blood circulation which could be switched to positive in response to external stimuli such as tumor extracellular pH (pHe), could be utilized to coat positive-charge NPs to obtain ideal nano-vehicles for effective delivery 27. In addition, the effective intracellular cargo release of NPs is crucial to achieve anticipated therapeutic effect. Intracellular stimuli such as redox environment different from the blood have been used to achieve on-demand and effective payloads release of the corresponding stimuli-responsive NPs 28.

**Scheme 1:**
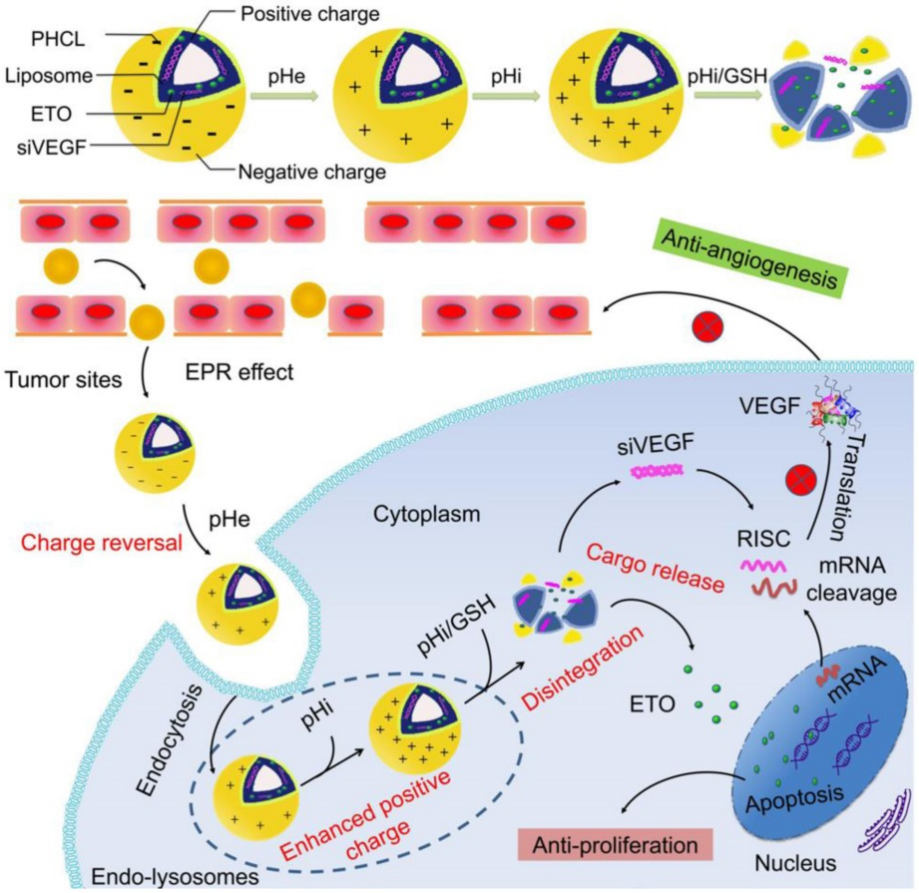
Schematic Illustration of Co-delivery of VEGF siRNA and Etoposide for Enhanced Anti-angiogenesis and Anti-proliferation *via* Multi-functional Nanoparticles in Orthotopic Non-Small Cell Lung Cancer Treatment *in vivo.*

Given the above challenges, we applied multi- component integration concept to construct pH/ redox responsive co-delivery nanoparticles for ETO and siVEGF to achieve effectively combined anti- proliferation and anti-angiogenesis therapy for orthotopic NSCLC. This regimen has not been reported previously. In this study, we modified previously reported versatile polymer histidine-grafted chitosan- lipoic acid (HCL) with PEGylation (PHCL) to coat cationic Lip via electrostatic adsorption and hydrophobic interaction and constructed multi- functional co-delivery vesicles PHCL-Lip 29. The cationic Lip incorporated siVEGF and ETO while pH-triggered charge-controllable and redox-responsive PHCL polymer played “guard and soldier” during the delivery process. As shown in Scheme 1, first, histidine segment in PHCL polymer enabled co-delivery nanoparticles (PHCL-Lip/ETO- siVEGF) with negative charge, which could shield the strong positive charge of cationic Lip as “guard” to improve stability in blood circulation and avoid the adverse effect on normal tissues. After accumulation in the tumor tissues via nanosize-induced EPR effect, weakly acidic surroundings (extracellular pH 6.5) triggered protonation of the imidazole group in histidine segment of PHCL leading to charge reversal of co-delivery vesicles from negative to positive as “soldier”, which improved penetration into the deep tumor and enhanced cell internalization. Subsequently, low pH in endo-lysosome triggered a stronger positive charge of PHCL layer and phase transition of DOPE component of Lip synergistically induced effective endosomal escape of payloads. Afterward, intracellular redox-responsiveness further triggered effective and sharp active cargo release and subsequently led to ETO-induced tumor cell apoptosis and siVEGF-induced VEGF signal pathway blocking. Thus, the co-delivery system developed in this study simultaneously suppressed tumor growth and blocked tumor neovascularization. The combined anti-proliferation and anti-angiogenesis therapeutic efficacy of PHCL-Lip-based efficient co-delivery of siVEGF and ETO for orthotopic NSCLC was systemically and comprehensively researched *in vitro* and* in vivo*.

## Materials and Methods

### Preparation and characterization of PHCL-Lip/ETO-siVEGF NPs

The cationic Lip was fabricated by emulsification solvent evaporation method. In brief, DOTAP, DOPE, and cholesterol with a weight ratio of 1:1:0.5 were added and ultrasonically dispersed, followed by rotary evaporation at 60 °C and filtered through 0.45 μm membranes to obtain blank Lip. Similarly, ETO- loaded Lips (Lip/ETO) were prepared with the addition of 0.2 mL of ETO solution (10 mg/mL) to Lips before ultrasonication. Subsequently, siVEGF was loaded by electrostatic adsorption with an optimal N/P ratio to obtain Lip/ETO-siVEGF. The final PHCL-Lip/ETO-siVEGF was obtained by incubating Lip/ETO-siVEGF with the PHCL layer solution. DSPE-PEG was added to prepared Lip/ETO-siVEGF to obtain P-Lip/ETO-siVEGF 30. The hydrodynamic diameter and zeta potential of blank cationic Lips (Lip), blank PHCL NPs (PHCL), PHCL coated Lips (PHCL-Lip) and PEGylated Lips (P-Lip) under different pH or 10 mM GSH incubation condition were measured using a ZEN 3600 (Malvern, UK). The morphology of PHCL-Lip/ETO under different pH and redox conditions was observed through a transmission electron microscopy (TEM, JEOL Ltd., Japan).

### Stimuli-responsive release of ETO from NPs

*In vitro* release behavior of stimuli- responsive NPs was investigated via the dialysis method. Briefly, formulations samples (3 mL) with ETO concentration of 0.2 mg/mL were transferred into dialysis bags (MWCO 3500 Da) and dialyzed against PBS (pH 7.4 or 5.3 with or without 10 mM GSH containing 10% ethanol) at 37 °C with 100 rpm. At predesigned time points, 1 mL release medium were taken and supplemented with 1 mL fresh medium. The concentration of ETO was measured by high performance liquid chromatography (HPLC) method with a ultra-violet wavelength of 285 nm and Thermo^®^ C18 column (250 × 4.6 mm, 5 μm) with methanol and water (70:30, v/v) as the mobile phase and a flow rate of 1 mL/min.

### Cell internalization and intracellular distribution

The fluorescent dye Nile Red (NR) and siRNA^FAM^ were used to trace non-fluorescence ETO and siVEGF, respectively. Luc-A549 cells were seeded (4×10^5^ per well) and incubated for 24 h. After treatment with PHCL-Lip/NR-siRNA^FAM^ or P-Lip/NR- siRNA^FAM^ at pH 7.4 or 6.5 for 2 h, cells were harvested and rinsed with pre-cooled PBS several times. The fluorescence intensity was measured by flow cytometry analysis (FC500, Beckman Coulter, USA).

The intracellular distribution of various formulations was monitored by confocal laser scanning microscopy (CLSM) (ZEISS, Germany). In brief, cells were incubated with PHCL-Lip/NR- siRNA^FAM^ or P-Lip/NR-siRNA^FAM^ at pH 7.4 or 6.5 for 2 or 6 h (NR 5 μg/mL and siRNA^FAM^ 100 nM). Nuclei were stained with Hoechst33258 and cells were imaged by CLSM. Cells treated with PHCL-Lip/ siRNA^FAM^ or P-Lip/ siRNA^FAM^ at pH 7.4 or pH 6.5 were also examined to study the co-location of siRNA^FAM^ with endo- lysosome stained with Lysotracker-red^®^.

### Evaluation of VEGF silencing efficacy

*In vitro* VEGF downregulation effects of various Lip formulations were evaluated by RT-PCR assay and Western blot analysis. Briefly, Luc-A549 cells (5×10^5^/well) were seeded into 12-well plates followed by 24 h of incubation. Cells were subsequently exposed to serum-free medium containing different formulations (the final siVEGF concentration 100 nM) for 6 h. The culture medium was then replaced with fresh complete medium followed by further 48 h of incubation. The cells and the medium were collected for further analysis. Total RNA was extracted for the RT-PCR analysis according to the standard protocol to observe VEGF downregulation at the mRNA levels. Also, the total protein in different groups was extracted and analyzed by Western blotting to monitor VEGF protein expression.

### Evaluation of cytotoxicity by MTT assay

The *in vitro* cytotoxicity of various Lip formulations was evaluated using the MTT assay. Luc-A549 cells were seeded into 96-well plates (5000 cells/well) followed by 24 h of incubation. After exposure to 200 μL of PBS (pH 7.4 or 6.5) containing different ETO/siVEGF formulations for 6 h, cells were further incubated with fresh complete medium for another 48 h. Cell viability was measured by MTT assay. Briefly, the absorbance of each group was determined by a full wavelength microplate reader (Infinite M1000 PRO, TECAN, Switzerland) at 490 nm**.** Cell viability was calculated via the following formula: Cell viability = (A_490(sample)_ - A_0_)/(A_490(control)_ - A_0_)×100 where A_sample_ and A_control_ represent the absorbance of sample formulations treated cells group and non-treated cells group, respectively. A_0_ represents medium-only group. The cell killing ability of blank Lip, layer PHCL, P-Lip and PHCL-Lip against Luc-A549 cells and human normal lung epithelial cells BEAS-2B were also measured.

### Penetration of NPs into Luc-A549 tumor spheroids

Three-dimensional (3D) spheroids model of Luc-A549 cells *in vitro* was established to investigate the tumor penetration capacity of PHCL-Lip formulations. Briefly, agarose solution (2%, w/v) was prepared and added to the bottom of the 96-well plate to form grooves. A549 cells were seeded in the grooves with 600 cells per well and cultured for 7 days to form tumor spheroids. The spheroids were removed from the wells and exposed to PHCL-Lip/ NR or P-Lip/NR at pH 7.4 or 6.5 for 2 h, followed by scanning fluorescent images via the Z-stack function of CLSM.

### *In vivo* targeting efficiency and biodistribution in orthotopic NSCLC

DiR was used as the fluorescence tracing dye and DiR-loaded NPs were prepared with the method described above. The nude mice bearing orthotopic Luc-A549 tumor were intravenously administrated with PHCL-Lip/DiR or P-Lip/DiR (DiR concentration was 300 μg/kg). 12 h or 24 h post administration, Luc and DiR fluorescent images were captured via IVIS Spectrum (Caliper science). Mice were sacrificed, and the major organs were excised for further imaging. Fluorescence intensity of *in vivo* or *ex vivo* images of different groups was acquired by the ROI analysis of the individual channels. The biodistribution of two formulations was also studied. In brief, 24 h after the administration of PHCL-Lip/NR or P-Lip/NR (NR concentration was 13.5 mg/kg), mice were sacrificed and the major organs were harvested. After homogenization, the NR was extracted and the fluorescence intensity was detected by a full wavelength microplate reader at the excitation wavelength of 510 nm and the emission wavelength of 583 nm. The concentration of NR was calculated using a standard curve.

### *In vivo* anticancer efficacy

To evaluate the *in vivo* antitumor efficacy and systemic toxicity of the co-delivery nanoparticles, nude mice bearing orthotopic Luc-A549 tumor model were randomly assigned to 6 groups with 5 mice in each group and administrated intravenously via tail vein with saline, ETO, P-Lip/ETO-siVEGF, PHCL- Lip/ETO, PHCL-Lip/siVEGF and PHCL-Lip/ETO- siVEGF (siVEGF= 1 mg/kg and ETO= 15 mg/kg), respectively. Mice received a total of 5 doses with each dose given every 3 days. The Luc fluorescence intensity was reported to be positively correlated to orthotopic tumor volume in our previous study 31. The bioluminescence in mice was monitored via IVIS Spectrum every 5 days. Body weight of mice was monitored every 2 days. After 30 days, mice were sacrificed, and the major organs were excised for immediate imaging and further immunofluorescence analysis. Besides, *H&E* staining was used to further study the antitumor efficacy and side effects.

### Statistical analysis

All the data in this paper were presented as mean ± standard deviation (SD) and statistical comparison were conducted by ANOVA analysis. Student's test was performed for pairwise comparison. Statistically significant difference was defined as * *p*< 0.05, ** *p*< 0.01 and *** p< 0.001, respectively.

## Results and discussions

### Synthesis and characterization of PHCL layer

The functional layer PHCL polymer was obtained by modifying previously reported HCL polymer with mPEG via amidation reaction, following the route shown in Figure. S1A. The structure of PHCL was characterized by ^1^H NMR (shown in Figure. S1B). The signals at δ 4.26 ppm and 3.20-4.00 ppm referred to the CS structure (H_1_ and sugar ring). Compared with CS, the proton signals at 2.75, 3.10 and 3.75 ppm referred to the proton peak of penta-heterocycle, and δ 1.31, 1.50, 1.75 and 2.55 ppm referred to the -CH_2_- in lipoic acid (LA), which demonstrated the introduction of LA to CS. Similarly, the proton signals at 7.70 ppm and 6.84 ppm belonging to the proton peaks of the imidazole group in histidine (His) confirmed the successful synthesis of HCL. Finally, PEGylation of HCL polymer was demonstrated by the combinational proton signals at 3.45 ppm (CH_3_O-) and 3.68 ppm (-CH_2_-). The amount of LA, His, and mPEG in PHCL were calculated to be 15.1%, 32.0%, and 20.2%, respectively.

### Preparation and characterization of coated NPs

**Figure 1:**
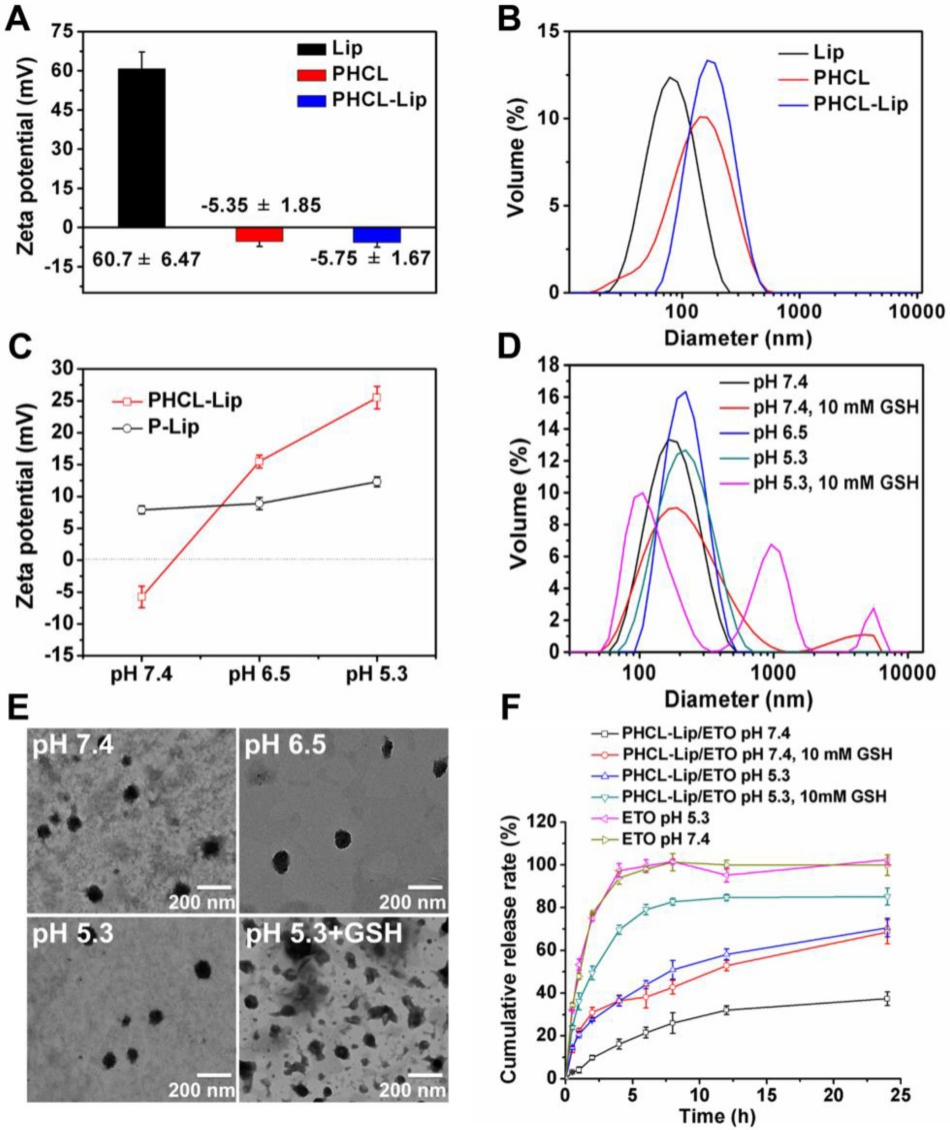
(A) Zeta potential and (B) particle size of the blank Lip, PHCL NPs and PHCL-Lip; (C) Zeta potential of PHCL-Lip and P-Lip at different pH; (D-E) The size distribution and TEM images of PHCL-Lip under different pH and redox conditions; (F) *In vitro* ETO release profiles from PHCL-Lip/ETO and ETO injection when exposed to different pH and redox stimuli.

The charge reversal and redox-responsive property of blank PHCL-Lip responding to the stimuli of pH and redox was evaluated. As shown in Figure 1A, compared with strong positively charged Lip, PHCL- Lip showed a negative charge of -5.75 mV close to PHCL nanoparticles. Besides, PHCL-Lip exhibited a bigger diameter than Lip and PHCL with a single peak in distribution (Figure 1B), indicating that cationic Lip was successfully coated with PHCL layer. Noticeably, as the pH condition dropped from 7.4 to 6.5, the *zeta* potential of PHCL-Lip reversed from the negative charge of -5.75 mV to a positive charge of +15.5 mV and exhibited further strong positive charge of +25.5 mV at pH 5.3 (Figure 1C). On the other hand, DSPE-PEG modified liposomes (P-Lip) had no significant difference in zeta potential as the surrounding pH value changed. PHCL-Lip with negative charge in physiological pH showed higher stability and less interaction with blood components (Figure S2 and Figure S4A) than the positively charged P-Lip 32. Besides, the size distributions of PHCL-Lip exposed to different pH values (Figure 1D) revealed that PHCL-Lip had a uniform size of 161.3 nm at pH 7.4 and exhibited an increase in size as pH value further decreased. Significantly, exposure to pH 5.3 and 10 mM GSH resulted in multipeak phenomenon and much broader size distribution of PHCL-Lip, which might be attributed to instability and even disintegration caused by pH-triggered protonation of the imidazole group of His segment and redox-activated breakage of disulfide bond of LA segment in PHCL-Lip. These results were further demonstrated in Figure 1E. PHCL-Lip presented uniform size distribution at pH 7.4. Remarkable destabilization and aggregation were observed after treatment with 10 mM GSH. These results indicated that PHCL-Lip with negative charge in the physiological environment might improve the stability in blood circulation and then promote internalization by tumor cells through charge reversal after accumulation in tumor sites *via* EPR effect with an appropriate nanosize. Notably, the destabilization and rupture of coated nanoparticles in response to intracellular pH/redox stimuli might facilitate the on-demand intracellular sharp cargo release.

### *In vitro* stimuli-responsive payload release

The effective and on-demand intracellular drug release is critical for desired therapy efficacy, thus the *in vitro* drug release behavior of different ETO formulations responding to pH and GSH stimuli was examined. PHCL-Lip/ETO-siVEGF was prepared and the optimal formulation with 176.5 nm, - 12.06 mV and excellent cargo loading capacity was obtained (Table S1 and Figure S3). Additionally, PHCL-Lip NPs significantly protected siVEGF from nuclease degradation (Figure S4B). Figure 1F revealed that PHCL-Lip/ETO performed a sustained release with 37.4 % of ETO released at 24 h. Furthermore, PHCL- Lip/ETO exhibited similar release behavior at pH of 6.5 and 7.4 (Figure S5A). However, pH 5.3 and 10 mM GSH markedly stimulated sharp and almost complete ETO release from PHCL-Lip/ETO up to 80% at 6 h. The ETO release from P-Lip/ETO showed no noticeable difference in the presence of 10 mM GSH (Figure S5B). The release at pH 5.3 was higher than that at pH 7.4, which might be due to the pH sensitivity of DOPE in cationic liposomes 33. Besides, PHCL-Lip/ETO exhibited relatively slower drug release than P-Lip/ETO at pH 7.4, indicating that PHCL layer might protect against cargo leakage. It was likely that the sustained and slow ETO release from PHCL-Lip/ETO in blood circulation but sharp and sufficient release after internalization by tumor cells would reduce the side effects of ETO to normal tissues and quickly attain high enough intracellular free ETO concentration to enhance tumor cell killing ability.

### Cellular uptake and intracellular translocation

**Figure 2:**
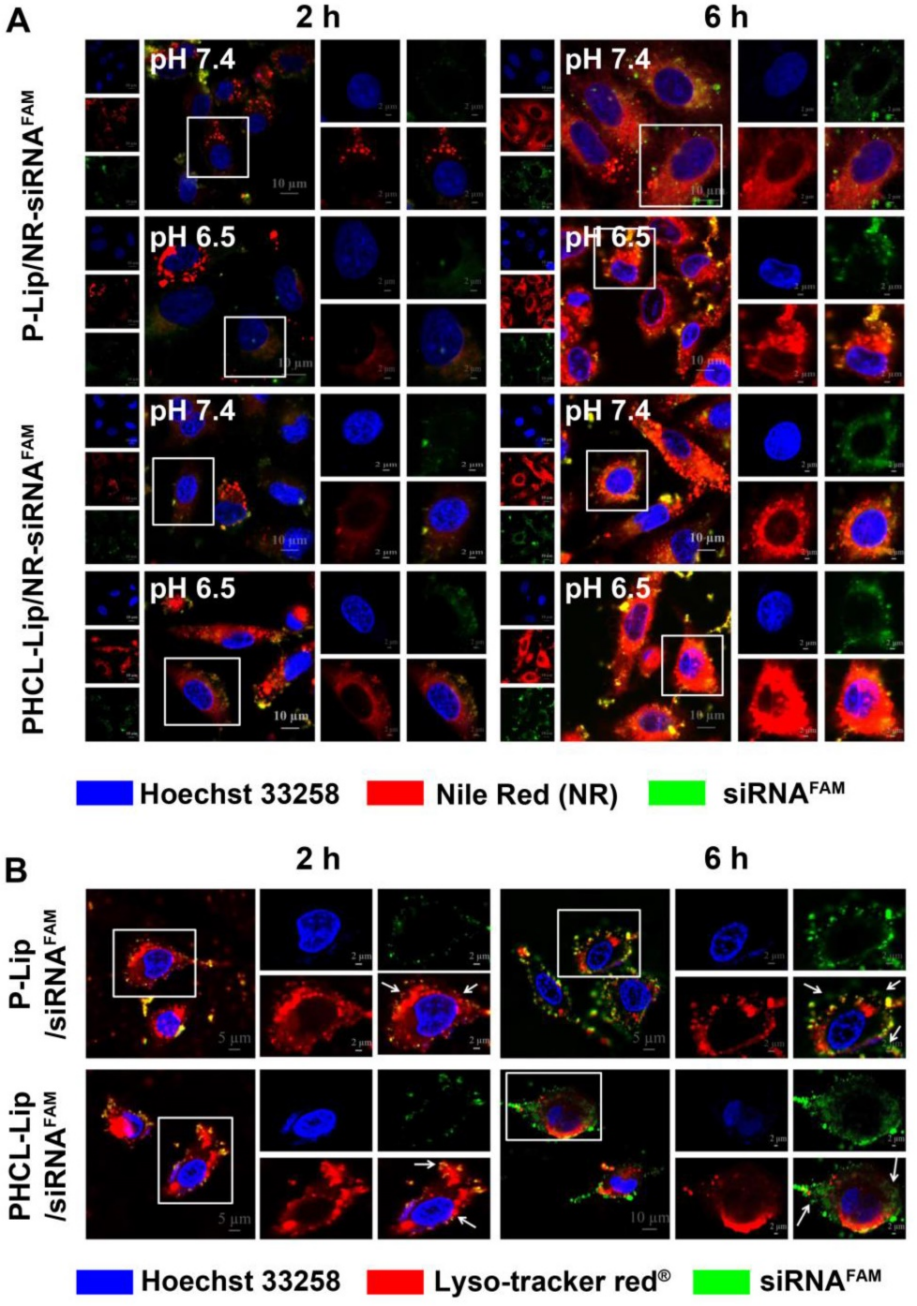
(A) Cellular uptake and intracellular distribution of different formulations. CLSM images of A549 cells treated with (A) P-Lip/NR-siRNA^FAM^ or PHCL-Lip/NR-siRNA^FAM^ at pH 7.4 or 6.5 for 2 or 6 h and (B) P-Lip/siRNA^FAM^ or PHCL-Lip/siRNA^FAM^ at pH 6.5 for 2 or 6 h.

Sufficient drug concentration is critical for desired therapeutic efficacy which requires efficient internalization of drug-loaded NPs followed by sharp and on-demand cargo release within cells 34. We chose the NR dye to trace non-fluorescent drug ETO. First, quantitative uptake of PHCL-Lip/NR- siRNA^FAM^ under different pH conditions was evaluated by flow cytometry with P-Lip/NR-siRNA^FAM^ as comparison. As shown in Figure S6, PHCL-Lip/NR- siRNA^FAM^ exhibited significantly enhanced cellular uptake at pH 6.5 than that at pH 7.4 attributed to the positive charge of nanoparticles triggered by pH 6.5. The pH change made no discernable difference for cell internalization of P-Lip/NR-siRNA^FAM^. Subsequently, the cellular uptake and intracellular distribution were observed by CLSM. As is evident from Figure 2A, PHCL-Lip/NR-siRNA^FAM^ showed significantly stronger red and green fluorescence intensity at pH 6.5 than at pH 7.4 while no obvious difference was observed between P-Lip/NR-siRNA^FAM^ at both pH values. This observation was consistent with the results of flow cytometry analysis. Thus, it appeared that PHCL layer enhanced cellular uptake of the NPs through pHe-triggered charge reversal and facilitated the release of NR in response to intracellular acidic and redox environment, which would guarantee enough intracellular drug concentration and produce an advanced antiproliferation effect on tumor cells.

In the case of siRNA, it is critical to achieve adequate active siRNA to targeted mRNAs in the cytoplasm through effective endo-lysosomal escape. The distribution of siRNA^FAM^-loaded formulations within cells after internalization was investigated (Figure 2B). At 2 h, there was complete co-location of green fluorescence of siRNA with the red fluorescence of endo-lysosomes in both PHCL-Lip and P-Lip groups. Notably, at 6 h, PHCL-Lip group showed remarkable non-overlap between the green fluorescence of siRNA and red fluorescence of endo-lysosome than the P-Lip group. It appeared that the endo-lysosomal pH-triggered positive charge increase in the PHCL layer improved endo-lysosome escape capability of PHCL-Lip NPs preventing siRNA from inactivation and leading to enhanced gene silencing effect.

### VEGF downregulation efficacy of NPs

**Figure 3:**
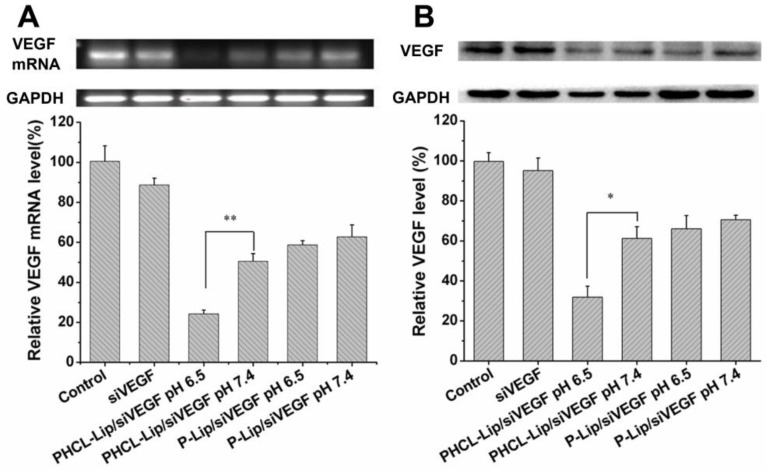
(A) VEGF mRNA level of A549 cells by RT-PCR analysis and (B) VEGF protein expression level in the culture medium of A549 cells by Western blot after treated with various siVEGF formulations.

siRNA has been reported to silence specific gene *via* incorporating into RNA-induced silencing complex (RISC) and caused degradation of targeted mRNAs and subsequent downregulation of specific protein. The concentration of siVEGF in various formulations used in this study was 100 nM. Compared with Lipofectamin 2000, PHCL-Lip demonstrated almost equivalent transfection efficiency (Figure S7). As shown in Figure 3A, 2.08- fold stronger downregulation of VEGF was observed in PHCL-Lip/siVEGF group at pH 6.5 than at pH 7.4. There was no significant difference for P-Lip/siVEGF group as pH changed. Also, PHCL-Lip/siVEGF at pH 6.5 showed approximately 1.92-fold downregulation of VEGF at the protein level compared with pH 7.4 by Western blotting (Figure 3B). Results indicated that extracellular pH significantly advanced VEGF silencing effect of PHCL-Lip formulations due to enhanced cellular uptake and improved endosomal escape.

Angiogenesis has been reported to play a significant role in promoting tumor proliferation and metastasis. VEGF is a well-known pro-angiogenic factor produced by tumor cells that significantly induces tumor angiogenesis by promoting proliferation of endothelial cells 35. Silencing VEGF expression blocks VEGF-VEGFR signaling in the endothelial cells of tumor blood vessels 36. Hence, the effect of VEGF downregulation on proliferation of human umbilical vein endothelial cells (HUVEC) was further investigated by MTT assay. As shown in Figure S8, the conditioned medium of PHCL-Lip/ siVEGF (pH 6.5)-treated A549 cells exhibited the weakest growth promoting effect on HUVEC which indicated that PHCL-Lip/siVEGF could effectively suppress the formation of blood vessels in mildly acidic tumor sites. It was also reported that VEGF might promote HUVEC migration and invasion and induce new tumor blood vessels network formation at other sites 37. Here, we investigated whether VEGF secretion from A549 cells transfected with PHCL-Lip/ siVEGF or P-Lip/siVEGF NPs influenced migration and invasion of HUVEC by using wound-healing and matrigel invasion assays. We found that the conditioned medium of cells treated with PHCL-Lip/ siVEGF could effectively weaken the migration and invasion ability of HUVEC, especially at slightly acidic environment (Figure S9), compared with other formulations. This could be due to the higher VEGF downregulation ability of PHCL-Lip/siVEGF NPs at pH 6.5.

### *In vitro* cytotoxicity study

**Figure 4:**
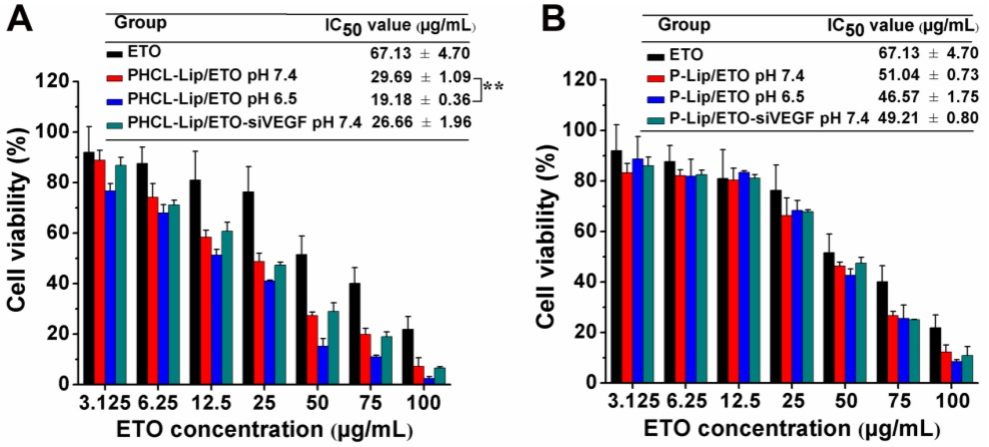
(A-B) Cytotoxicity investigation of various ETO or siVEGF formulations against A549 cells by MTT assay (n=5), wherein “*” indicated comparison between PHCL-Lip/ETO at pH 7.4 and 6.5.

The cell killing ability of various ETO formulations was investigated by MTT assay. When the cytotoxicity of various blank nanoparticles against A549 cells and human normal lung epithelial BEAS-2B cells was measured, we observed that the PHCL layer significantly improved the biocompatibility of the NPs (Figure S10). Figure 4 showed the pH-dependent anti- proliferation effect of PHCL-Lip/ ETO with 1.55-fold lower IC_50_ value at pH 6.5 than at pH 7.4. Also, PHCL-Lip/ETO at pH 7.4 showed 2.26- and 1.71-fold higher cell killing activity than P-Lip/ETO and free ETO, respectively, at the same pH. Change in pH values showed no apparent influence on cytotoxicity of P-Lip. Moreover, no remarkable difference was observed after the co-delivery of siVEGF with ETO in PHCL-Lip formulation at pH 7.4 indicating that siVEGF mainly downregulated VEGF expression to block angiogenesis while exhibiting little cytotoxicity against tumor cells. These observations suggested that the PHCL layer endowed PHCL-Lip with greater cellular internalization ability leading to enhanced anti-proliferation efficacy at extracellular pH.

### Evaluation of tumor spheroid penetrating ability of NPs

**Figure 5:**
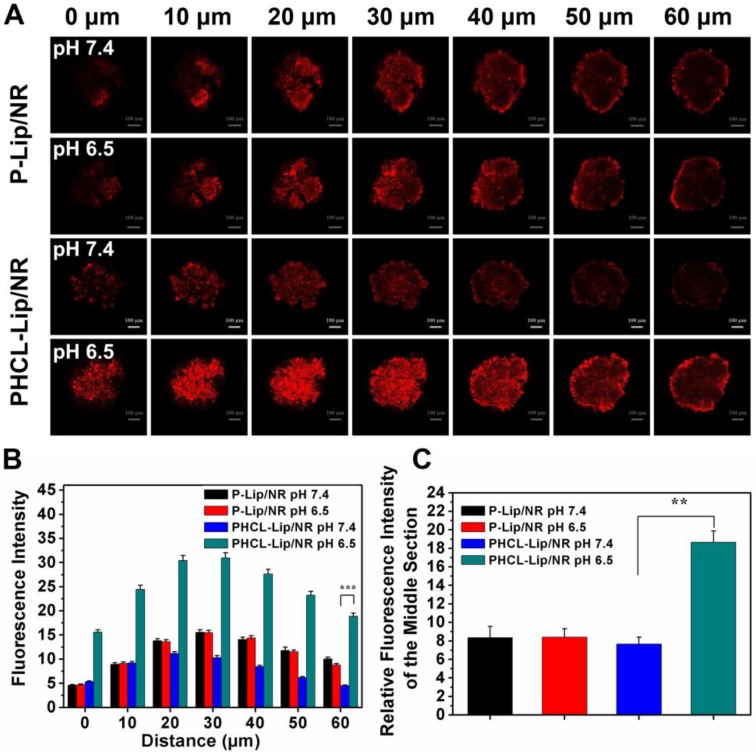
Penetration evaluation: (A) Scanned fluorescence distribution of the A549 spheroids after treated with PHCL-Lip/NR or P-Lip/NR at pH 7.4 or pH 6.5 conditions by CLSM. The concentration of NR was 5 μg/mL and bar was represented as 100 μm. (B) Semi-quantitative fluorescence intensity of different sections of A549 spheroids. (C) Semi-quantitated fluorescence intensity of center region of A549 spheroids at 60 μm section.

Adequate penetration of drug delivery systems into the internal region of tumors would largely improve the treatment 38. A549 tumor spheroids were constructed to investigate the *in vitro* tumor penetration of PHCL-Lip or P-Lip formulations. NR was used to trace ETO. The fluorescence distribution of different formulations in A549 tumor spheroids was shown in Figure 5A. Notably, PHCL-Lip/NR at pH 6.5 exhibited much stronger fluorescence intensity in all sections of spheroids and smaller fluorescence intensity decrease as the depth increasing than at pH 7.4. Semi-quantitative fluorescence intensity measurement presented in Figure 5B further confirmed the results. This might be due to the significant charge reversal of PHCL-Lip/NR in response to pHe stimuli, which promoted tumor penetration ability of NPs 39, 40. P-Lip/NR exhibited significantly lower tumor spheroids penetration than PHCL-Lip/NR at pH 6.5 with no significant difference between pH 6.5 and 7.4. Furthermore, the fluorescence intensity of the center region of A549 spheroids at 60 μm section was semi-quantitated in Figure 5C. PHCL-Lip/NR at pH 6.5 exhibited much stronger fluorescence than other groups. These results indicated that the PHCL-Lip formulation would penetrate into the center region of tumor due to the positive charge reversal in the acidic environment of tumor sites.

### Evaluation of* in vivo* targeting efficiency

**Figure 6:**
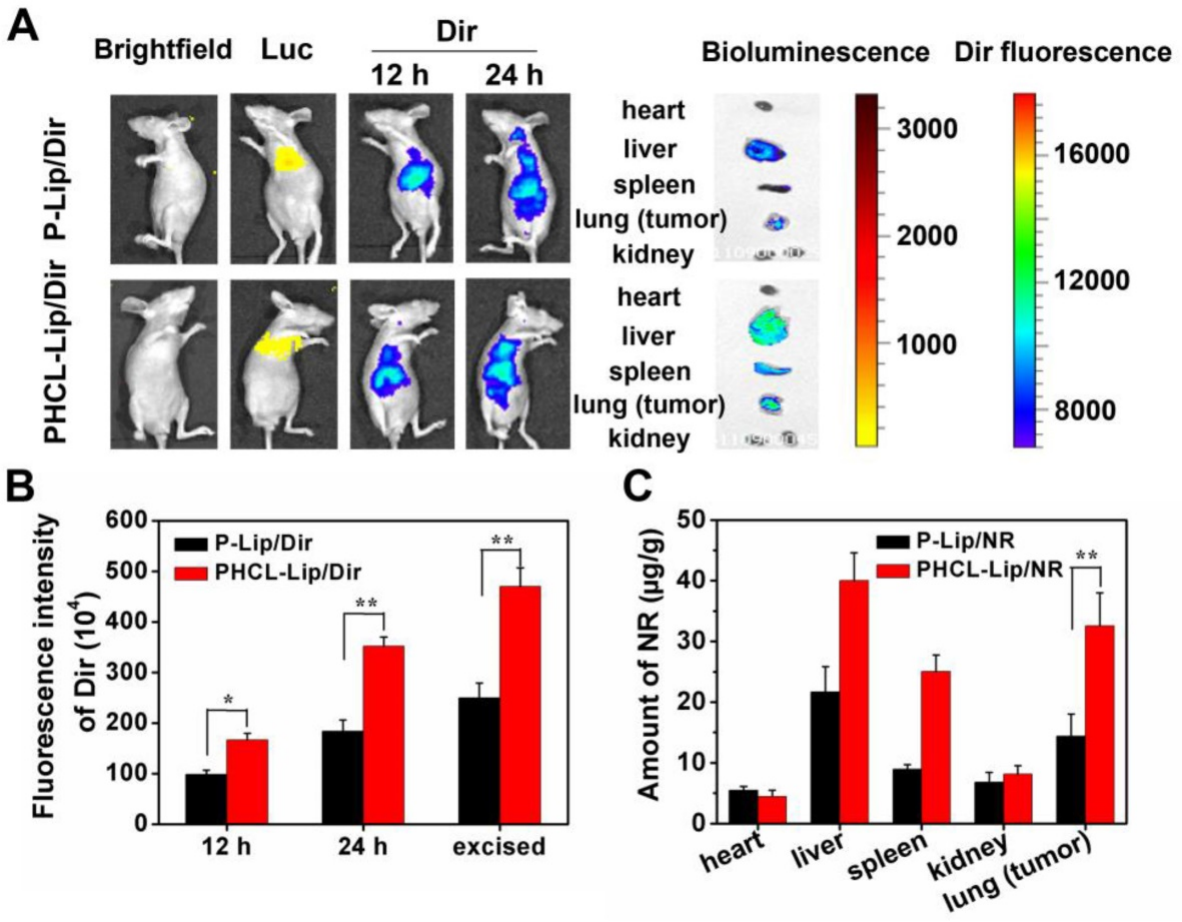
*In vivo* targeting evaluation and biodistribution of co-delivery formulations. (A) In vivo fluorescent and bioluminescent images of orthotopic A549 nude mice model after administrated with P-Lip/Dir or PHCL-Lip/Dir for 12 or 24 h; (B) Relative fluorescence intensity of the lung (tumor) of nude mice at 12 , 24 h or excised section; (C) Biodistribution of P-Lip/NR or PHCL-Lip/NR in orthotopic A549 nude mice after administration.

Efficient drug accumulation in tumor sites is critical for delivery systems to achieve the desired therapeutic effect and high safety *in vivo*. We established the orthotopic A549 nude mice model to evaluate the targeting efficacy of our multifunctional nanosystem (1.1 section in Supporting Information). Fluorescent dye DiR was selected to trace the *in vivo* distribution of different formulations. The *in vivo* DiR fluorescent and Luc bioluminescent images of mice at predesigned time points after intravenous injection of PHCL-Lip/DiR or P-Lip/DiR were obtained by IVIS spectrum (Caliper life science). As shown in Figure 6A and B, PHCL-Lip exhibited much stronger DiR fluorescence in the lung compared with the P-Lip group. The images of excised lungs confirmed these results, which were attributed to improved stability benefiting from charge-controllable PHCL layer leading to higher accumulation in tumor sites and subsequently enhanced cell internalization. Also, the stronger fluorescent signal observed in the excised liver of the PHCL-Lip group compared with P-Lip suggested that PHCL-Lip were stable in circulation while P-Lip suffered quick elimination because of the positive charge 41. Figure S11 suggested that these NPs were significantly accumulated in tumor lesion rather than normal lung tissues and PHCL-Lip exhibited better tumor targeting efficacy. The biodistribution study (Figure 6C) further demonstrated the above results indicating that PHCL- Lip possessed excellent targeting ability and adequate accumulation in tumor sites and may serve as ideal vesicles for cancer therapy.

### *In vivo* combined anticancer efficacy of siVEGF and ETO

**Figure 7:**
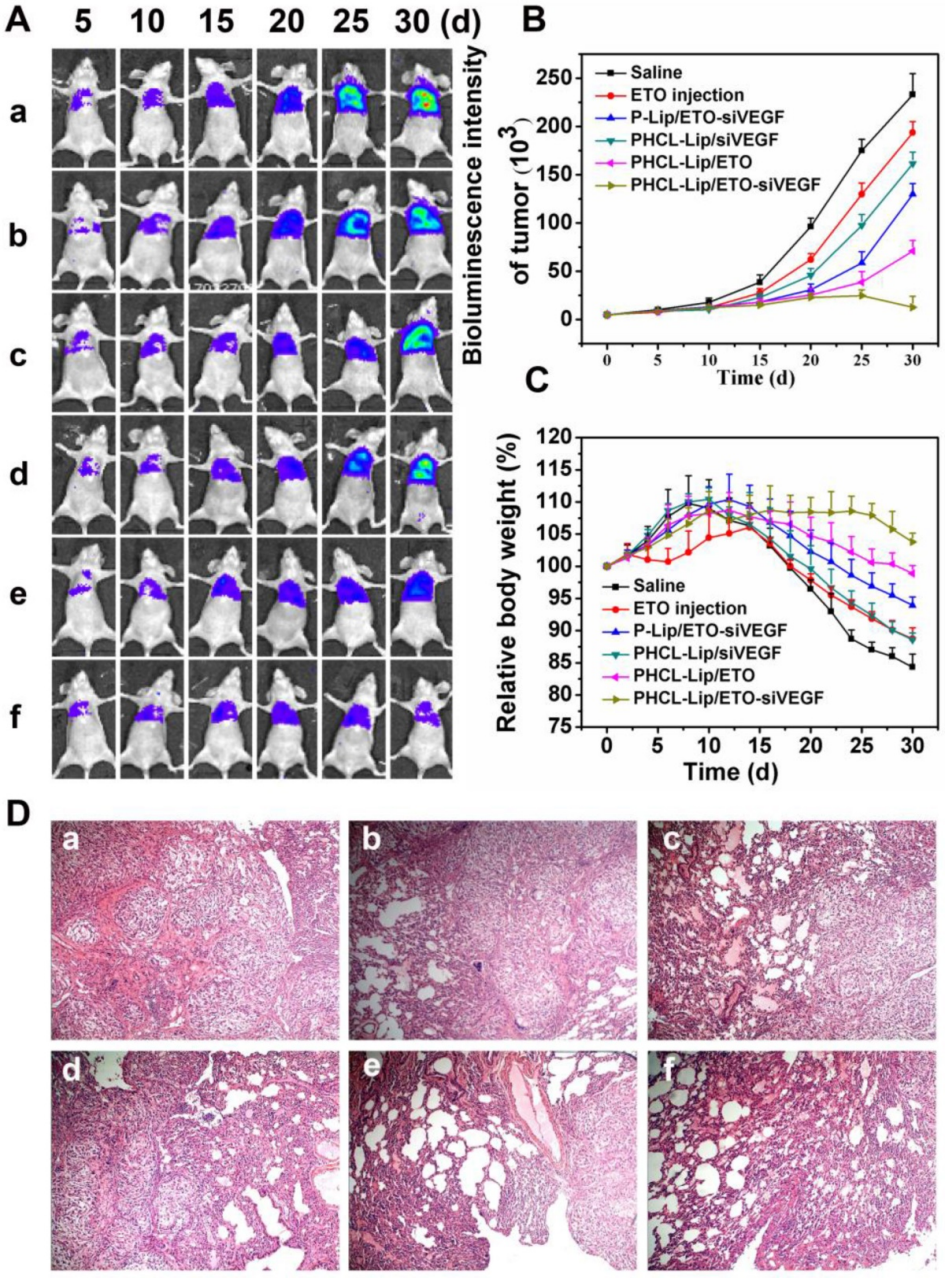
Tumor growth inhibition evaluations in orthotopic A549 tumor models. (A) Bioluminescence images and intensity (B) of mice treated with different formulations at predesigned time points; (C) Relative body weight of mice in different groups monitored every other day; (D) Representative *H&E images of orthotopic A549 lung tumors after* different formulations treatment. Different formulations represented a. Saline, b. ETO injection, c. P-Lip/ETO-siVEGF, d. PHCL-Lip/siVEGF, e. PHCL-Lip/ETO, f. PHCL-Lip/ETO- siVEGF. Data was represented as mean ± SD (n=5).

Massive angiogenesis and malignant tumor proliferation was found in NSCLC tumorigenesis and progression 42. The orthotopic tumor model was demonstrated to much closely recapitulate tumorigenesis, proliferation and metastasis in human than the subcutaneous tumor model 43, 44. Therefore, we evaluated the *in vivo* synergistic antitumor growth and anti-metastasis efficacy of co-delivery nanoparticles in the orthotopic A549 nude mice model for 30 days. The bioluminescence intensity of mice in predesigned time points was detected every five days to monitor tumor proliferation (shown in Figure 7A and B). Combined therapy with PHCL-Lip/ETO- siVEGF showed the most effective anti-proliferation efficacy. There was almost no noticeable increase in bioluminescence intensity during the treatment with even a decrease in the late phase of the treatment. However, monotherapy with PHCL-Lip/ETO or PHCL-Lip/ siVEGF exhibited temporary suppression of tumor growth at early treatment but showed insufficient antigrowth activity from approximately day 15 of the treatment. It was evident that combined therapy regimen possessed much higher and longer-term antitumor efficacy than monotherapy. P-Lip/ETO-siVEGF, on the other hand, revealed inadequate tumor suppression which might be due to its insufficient accumulation in tumor sites and relatively poor internalization. ETO injection alone had much lower inhibition than nano-delivery formulations.

The body weight of mice was monitored every other day (Figure 7C). Combined therapy was found to have little effect on body weight during the entire treatment. Combining the hematological analysis and blood chemistry test (Figure S12 and Figure S13), it was indicated that PHCL-Lip/ETO-siVEGF with desired antitumor activity had no apparent toxicity in mice. Hematoxylin and eosin (*H&E*) histopathology analysis of lung (Figure 7D) further demonstrated the excellent antitumor efficacy of PHCL-Lip/ETO- siVEGF with little tumor mass or visible tumor nodules.

**Figure 8:**
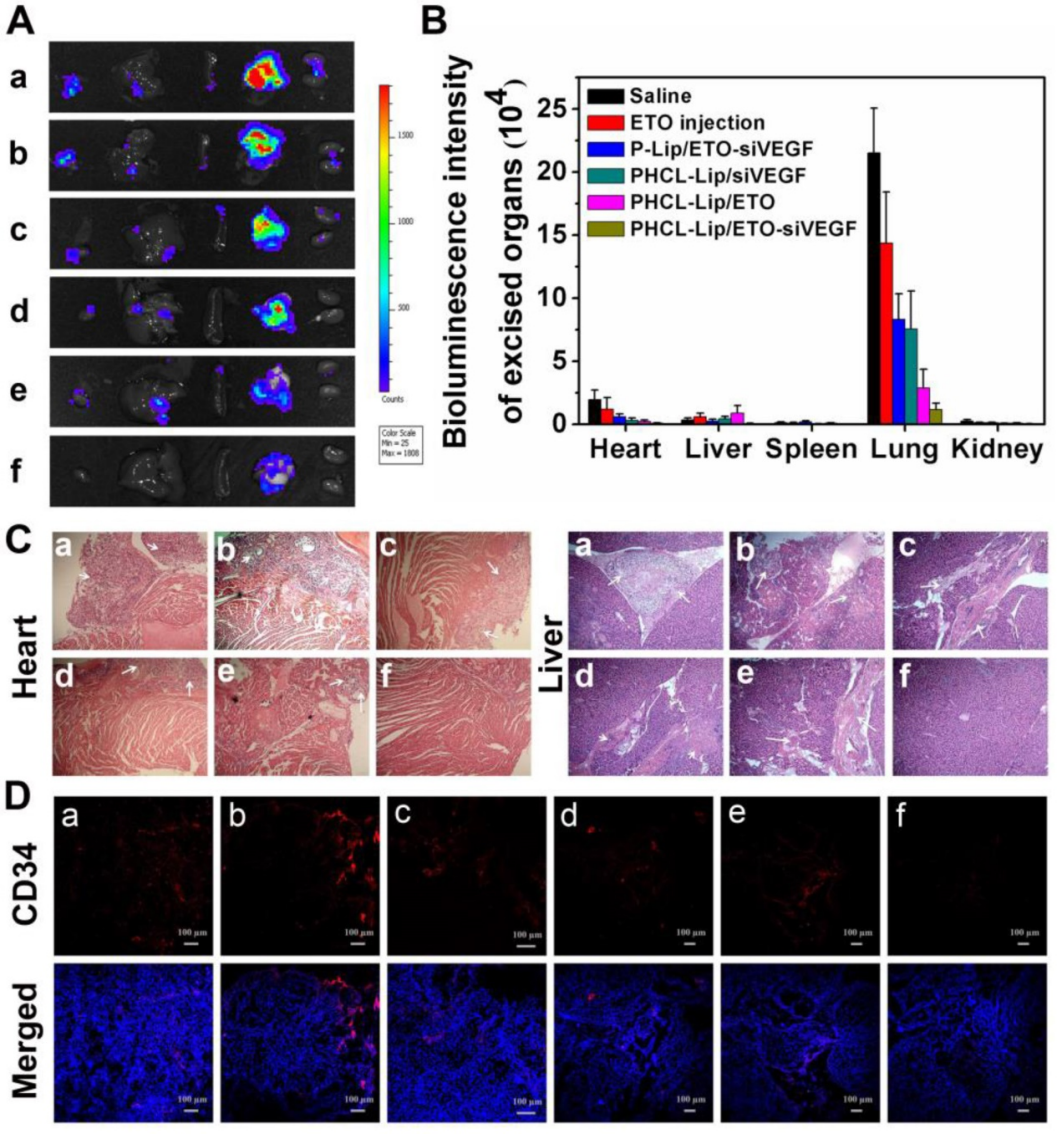
(A) Bioluminescence images and (B) intensity of excised major organs after treated with different formulations; (C) Representative *H&E images of heart and liver after* different formulations treatment; (D) Representative immunofluorescence images of vascular stained with blood vessel-specific-marker CD34 antibody in the orthotopic lung tumors of different groups. Different formulations represented a. Saline, b. ETO injection, c. P-Lip/ETO-siVEGF, d. PHCL-Lip/siVEGF, e. PHCL-Lip/ETO, f. PHCL-Lip/ETO- siVEGF. Data was represented as mean ± SD (n=5).

The bioluminescence images (Figure 8A) and fluorescence intensity (Figure 8B) of excised major organs of mice after treatment revealed tumor metastasis and distribution. Mice treated with saline or ETO exhibited visible bioluminescence in the heart and liver. Monotherapy of PHCL-Lip/siVEGF with insufficient inhibition of tumor proliferation showed weak metastasis bioluminescence in other organs which might be attributed to effective VEGF downregulation (Figure S14). Lower numbers of metastasis foci were observed in PHCL-Lip/ETO- treated group than saline and ETO injected group, resulting from effective tumor growth suppression and reduction in VEGF secretion. Significantly, combined therapy formulation of PHCL-Lip/ETO- siVEGF achieved the highest tumor metastasis inhibition. *H&E* histopathology analysis of the metastasized organs including the heart and liver (Figure 8C) showed that combined therapy exhibited almost no metastatic tumor masses. Furthermore, *H&E* analysis of spleen and kidney further confirmed that co-delivery nanoparticles possessed the desired safety *in vivo* (Figure S15).

We also examined the immunofluorescence images of blood vasculature staining with blood vessel-specific-marker CD34 antibody in the orthotopic lung tumors treated with different formulations by using CLSM. Figure 8D showed that combined therapy with PHCL-Lip/ETO-siVEGF exhibited a significant reduction in tumor vasculature which benefited from effective and combinatorial blocking of VEGF pathway and suppression of malignant proliferation. These results indicated that the combined therapy by effectively co-delivering ETO and siVEGF produced greatly enhanced inhibition of tumor growth and angiogenesis with lower systemic toxicity resulting in the desired antitumor efficacy of NSCLC *in vivo*.

## Conclusions

In this study, we successfully formulated a smart co-delivery system based on versatile nanoparticles for the combined application of ETO and siVEGF to simultaneously inhibit tumor growth and angiogenesis. The NPs utilized the multi-component concept to integrate cationic liposomes and stimuli- responsive PHCL polymer. These nanovehicles possessed the ability of on-demand effective co- delivery of ETO and siVEGF to orthotopic NSCLC. Compared with monotherapy, the combination therapy with ETO and siVEGF performed excellent and long-term inhibition of tumor proliferation and angiogenesis in orthotopic NSCLC with low biotoxicity. In conclusion, our study may promote the integration of anti-angiogenesis therapy and chemotherapy regimen for NSCLC treatment with significant potential for clinical translation.

## Supplementary Material

Supplementary figures and tables.
