# Supplementary material for: Co-delivery of VEGF siRNA and Etoposide for Enhanced Anti-angiogenesis and Anti-proliferation Effect via Multi-functional Nanoparticles for Orthotopic Non-Small Cell Lung Cancer Treatment
Source: Theranostics. 2019 Aug 12;9(20):5886–98. doi: 10.7150/thno.32416 (PMC6735374; doi:10.7150/thno.32416)
Supplement: Supplementary file 1 — Supplementary figures and tables. [file thnov09p5886s1.pdf]

Supplementary Materials

Co-delivery of VEGF siRNA and Etoposide for Enhanced Anti-angiogenesis and Anti-proliferation Effect *via* Multi-functional Nanoparticles for Orthotopic Non-Small Cell Lung Cancer Treatment

Fang Li[1,2*], Yu Wang[1*], Wei-liang Chen[1*], Dan-dan Wang[1], Ye-juan Zhou[1], Ben-gang You[1], Yang Liu[1], Chen-xi Qu[1], Shu-di Yang[1], Meng-tian Chen[1], Xue-nong Zhang[1]✉

1. Department of Pharmaceutics, College of Pharmaceutical Sciences, Soochow University, Suzhou 215123, People's Republic of China
2. Department of Pharmacy, Children` s Hospital of Soochow University, Suzhou 215025, People's Republic of China

*These three authors contributed equally to the paper.

✉ Corresponding author: The Department of Pharmaceutics, College of Pharmaceutical Sciences, Soochow University, DuShuHu High Education Zone, Su Zhou, Jiang Su Province, People's Republic of China, 215123; Tel/Fax: +86 (0512) 65882087; E-mail: zhangxuenong@suda.edu.cn

1. Experimental Section

1.1 Materials

VEGF siRNA (sense sequence: 5'- ACC UCA CCA AGG CCA GCA CTT - 3') and negative control siRNA (sense sequence: 5'- UUC UCC GAA CGU GUC AGG UTT -3') were synthesized by Shanghai GenePharma Co., Ltd. (Shanghai, China). Chitosan with molecule weight (MW) of 3-5 kDa and deacetylation degree of 95 % was purchased from 9 Ding Chemistry (Shanghai) Co., Ltd. *L*-Histidine was obtained from Sinopharm Chemical Reagent Co., Ltd. and *R*-alpha-Lipoic acid, 1-(3-dimethylaminopropyl)-3-ethyl carbon carbodiimide hydrochloride (EDC·HCl) and N-hydroxysuccinimide (NHS) were purchased from Adamas-beta-Reagent Co., Ltd. (Shanghai, China). Etoposide was gained from Shanghai Macklin Biochemical Co., Ltd. (Shanghai, China) and Methoxy PEG Succinimidyl Carboxymethyl Ester with MW of 2000 (M-SCM-2000) was purchased from JenKem Technology Co., Ltd. (Beijing, China), respectively. 1,2-Dioleoyl-3-trimethylammonium-propane chloride salt (DOTAP) and 1,2-Dioleoyl-sn-glycero-3-phosphethnalamine (DOPE) were obtained from Corden Pharma Switzerland LLC (Switzerland). Methyl thiazolyl tetrazolium bromide (MTT) was from Sigma-Aldrich (St. Louis, MO, USA). Hoechst

33258 was from Invitrogen (Eugene, OR, USA). Rabbit polyclonal VEGF antibody (A-20) was obtained from Santa Cruz Biotechnology, Inc. (Santa Cruz, CA, USA). RPMI-1640 medium was purchased from Hyclone Thermo-Fisher Biochemical Products Co., Ltd. (Beijing, China). Fetal bovine serum (FBS) was obtained from Zhejiang Tianhang Biotechnology Co., Ltd. (Hangzhou, China). Anti-CD34 antibody was purchased from Abcam (San Francisco, CA, USA). All other chemicals were of analytical grade and used without further purification.

Cell culture: Human non-small cell lung cancer (NSCLC) cell lines Luc-A549 and Human umbilical vein endothelial cells (HUVEC), originally obtained from Jiangsu Province Key laboratory of Biotechnology and Immunology (Suzhou, China), were cultivated in RPMI-1640 medium with 10% (v/v) FBS and 1% (v/v) penicillin−streptomycin solution. Cells were maintained in an incubator under 37 °C and 5% CO_2 condition.

Experimental animals: Female nude mice with age of 3-5 weeks, provided by the Experimental Animal Center of Soochow University (Suzhou, China), were used in all animals` studies. All mice were raised in the environment complying with the guidelines of the National Institute of Health for the care and use of laboratory animals. All animal procedures were performed following the protocols approved by the Institutional Animal Care and Use Committee. To establish the orthotopic lung cancer models, 5×10^6 A549 cells suspended in 100 μL of FBS-free medium containing matrigel (v/v=1:1) were injected through 1 ml-guage needle from about 1 cm down from the left armpit of nude mice under lateral position. The growth of the orthotopic tumor could be observed via IVIS Spectrum (Caliper science) after about one week. Each group was divided with 5 mice.

1.2 Synthesis and characterization of coated layer PHCL polymer

HCL polymer was obtained in our previous report.[1] PEGylation was modified into HCL polymer to obtain PHCL polymer. Briefly, methoxy PEG succinimidyl carboxymethyl ester (M-SCM-2000) and HCL polymer were mixed with molar ratio of 1:1 into 30 mL of distilled water, followed by further 24 h of stirring at room temperature under the catalysis of EDC·HCl and NHS. Afterwards, the mixture was

purified through dialysis (MWCO 1000) against distilled water for 48 h and PHCL polymer was then obtained via lyophilization. The successful synthesis of PHCL was confirmed by ^{1}H NMR spectroscopy (Varian, Palo Alto, CA, USA) at 400 MHz.

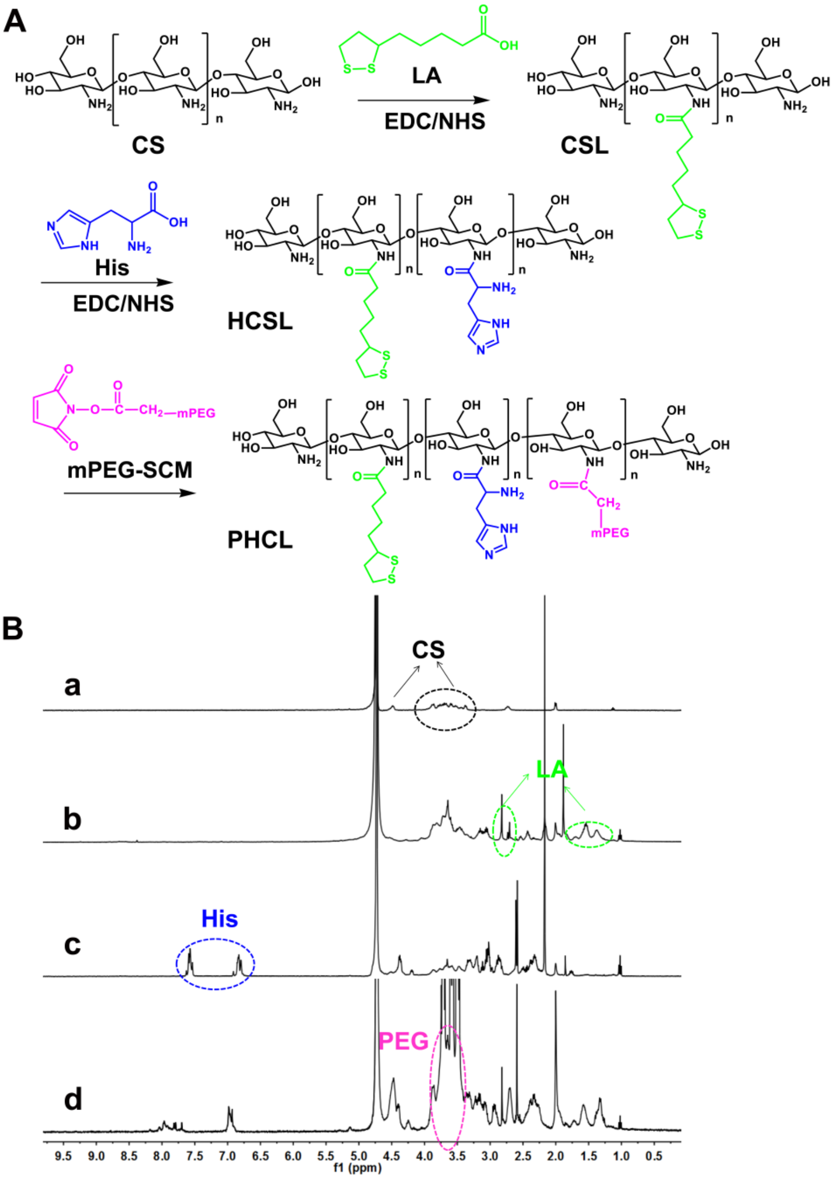

Figure S1. (A) Synthesis route of PHCL copolymer; (B) ^{1}H NMR spectrum of in D_2O of CS (a), CSL (b), HCSL (c) and PHCL (d).

1.3 Protein adsorption study

It is reported that surface charge of nanoparticles plays an important role in their stability and safety in circulation.[2]. For protein adsorption study[3], 100 μL of PHCL-Lip or P-Lip (1 mg/mL) was exposed to bovine serum albumin (BSA) solution with different pH for 6 h. The mixture solution was centrifuged with 30000 rpm at

4 °C for 15 min, followed by measuring the BSA concentration in supernatant. Protein adsorption (%) to the initially added BSA of different groups was shown in Figure S2B. PHCL-Lip at pH 7.4 revealed little protein adsorption. These results indicated that PHCL-Lip would show higher safety in vivo and less interaction with blood component.

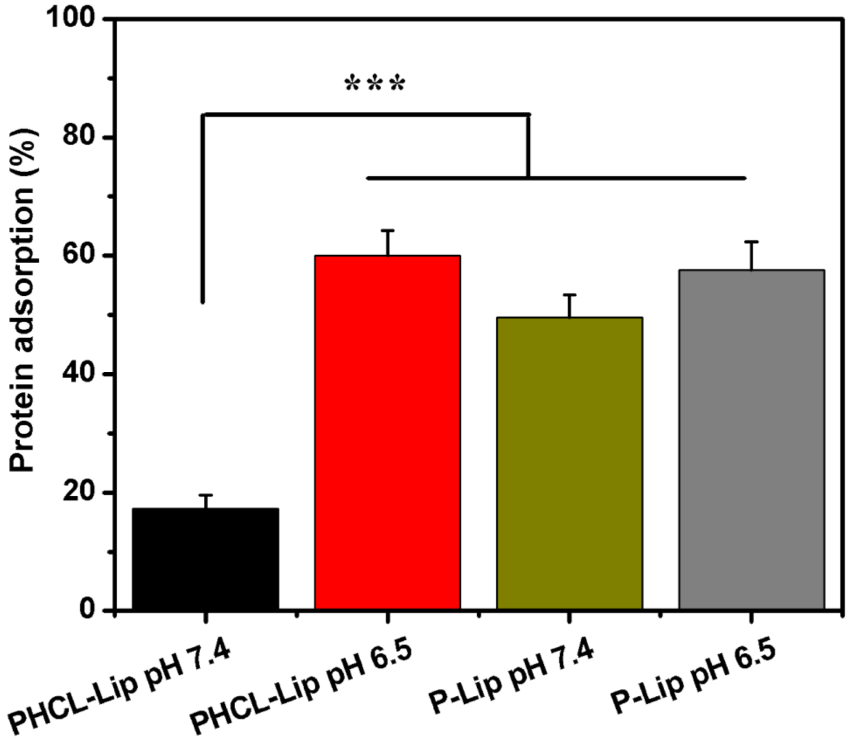

Figure S2. Protein adsorption of PHCL-Lip or P-Lip exposed to different pH for 6 h.

1.4 Preparation and characterization of ETO/siVEGF loaded NPs

Chemotherapeutic drug ETO and anti-angiogenic agent siVEGF were successively encapsulated into cationic liposomes with phospholipid bilayer and strong positive charge. Characterization of PHCL-Lip NPs and P-Lip NPs were shown in Table S1. A series of siVEGF-loaded Lip with P/N ratio of 1:0, 1, 2, 4, 6 and 8, wherein P was from siRNA and N was from DOTAP of cationic Lip, were formulated. The optimal N/P ratio was obtained via gel electrophoresis (shown in Figure S3). Besides, a series of siVEGF-loaded PHCL-Lip with Lip: PHCL (c/c) of 1:0, 1, 2, 4, 8 were prepared and characterized via gel electrophoresis to get optimal ratio of PHCL: Lip. Results in Figure S3 showed that the optimal prescription of PHCL-Lip nanoparticles was the N/P ratio of 6:1 and ratio of PHCL: Lip (w/w) of 1:1,

wherein PHCL-Lip nanoparticles could completely encapsulate added siVEGF with no leakage.

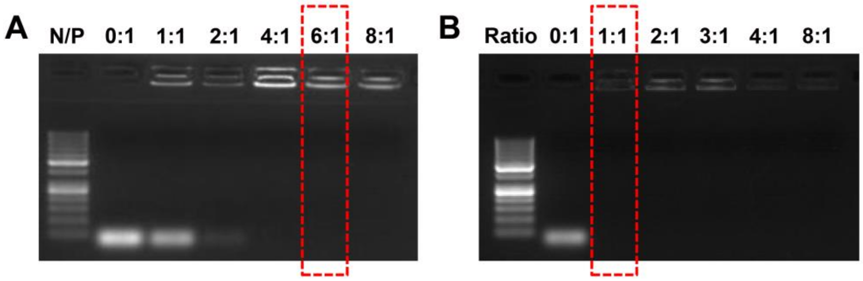

Figure S3. Optimal prescription screening. Gel electrophoresis image of siRNA/Lip with different N/P ratio (A) and siRNA/PHCL-Lip under optimal N/P ratio with different concentration ratio of PHCL: Lip.

1.5 Size stability and nuclease protection assay of NPs

Size stability of PHCL-Lip/ETO-siVEGF was studied under serum containing condition. In brief, 1 mL of PHCL-Lip/ETO-siVEGF was mixed with 1 mL of 10% serum. The mixture was then incubated at room temperature for 24 h. At predetermined time points, the particle size of PHCL-Lip/ETO-siVEGF was measured by a ZEH 3600 (Malvern, UK). The result was shown in Figure S4A. PHCL-Lip/ETO-siVEGF exhibited no significant increase in size during 24 h under serum containing condition, which indicated that PHCL significantly improved the stability of liposomes and PHCL-Lip/ETO-siVEGF would perform good stability *in vivo*.

Nuclease stability of siRNA encapsulated in NPs was significant for siRNA-based therapy. RNase protection assay and serum stability were investigated. PHCL-Lip/ETO-siVEGF was mixed with RNase (20 μg/mL) or 50% serum. The mixture was then incubated for predetermined time at 37 °C. Agarose gel electrophoresis was conducted to determine the remaining siRNA. Free siRNA was set as the positive control. As shown in Figure S4B, free siRNA was degraded by RNase within 2 h. And PHCL-Lip protected siRNA from RNase degradation for up to 48 h. Besides, PHCL-Lip/ETO-siVEGF also showed desired stability in 50% serum.

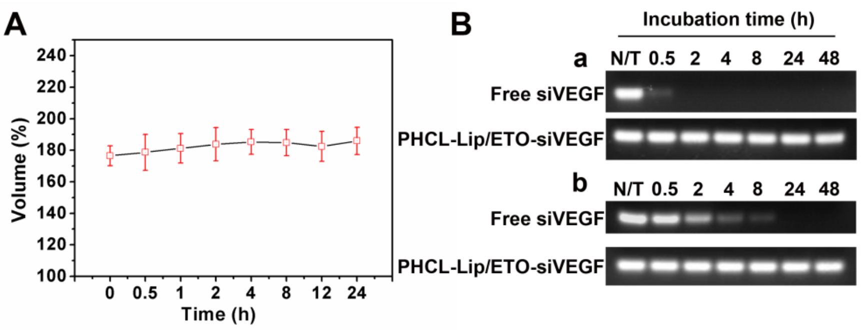

Figure S4. (A) Size stability of PHCL-Lip/ETO-siVEGF under serum containing conditions for 24 h; (B) Nuclease stability (a) or serum stability (b) of free siVEGF and PHCL-Lip/ETO-siVEGF; NT represented the non-treated samples.

1.6 The *in vitro* drug release of PHCL-Lip or P-Lip/ETO

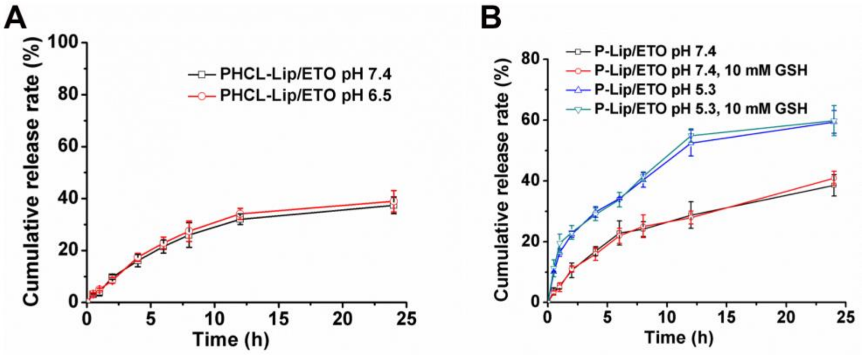

Figure S5. (A) The *in vitro* drug release of PHCL-Lip/ETO at pH 7.4 or pH 6.5; (B) The release behavior of P-Lip/ETO under different pH and redox environment.

1.7 Quantitative cell internalization analyzed by flow cytometry analysis

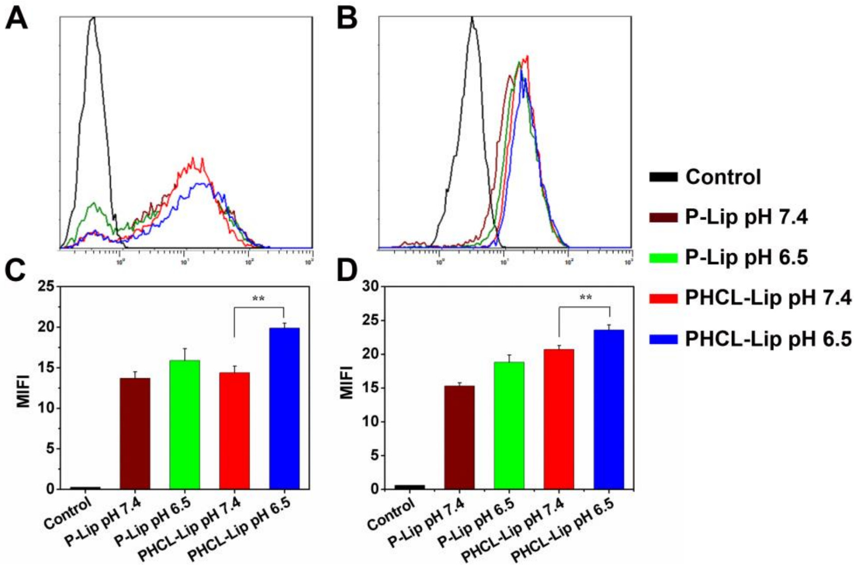

Figure S6. Quantitative uptake of different co-delivery formulations PHCL-Lip/NR-siRNA^{FAM} or P-Lip/NR-siRNA^{FAM} evaluated by FACS. Cell uptake curve of NR (A) and siRNA^{FAM} (B); Mean fluorescence intensity (MFI) of NR (C) and siRNA^{FAM} (D)

in cell with different treatments. Statistically significant difference was defined as * $p < 0.05$, ** $p < 0.01$ and *** $p < 0.001$, respectively.

1.8 RT-PCR and western blot analysis of PHCL-Lip/siVEGF and Lipofectamine 2000/siVEGF

RT-PCR and western blot analysis of PHCL-Lip/siVEGF and Lipofectamine 2000/siVEGF were conducted. For western blot analysis, the total protein of cell treated with different formulations was extracted and analyzed through western blot assay. In brief, after determine the concentration of protein using a BCA kit, a 40 μg of protein per group was separated by 10% SDS-PAGE and followed by transferred into polyvinylidene fluoride membranes. Then, the membranes were treated with 1× western block buffer in TBST containing 5% skimmed milk at room temperature for 1 h and subsequently incubated with rabbit affinity purified polyclonal VEGF antibody (Santa Cruze, CA, USA) at a dilution of 1:500 overnight at 4 °C. GAPDH antibody (1:10000 dilution) was selected as the internal control. After three times washing, the membranes were incubated with secondary antibody at a dilution of 1:1000 at room temperature for another 1 h. Finally, the membranes were imaged via a gel imager (ChemiDoc, BIO-RAD).

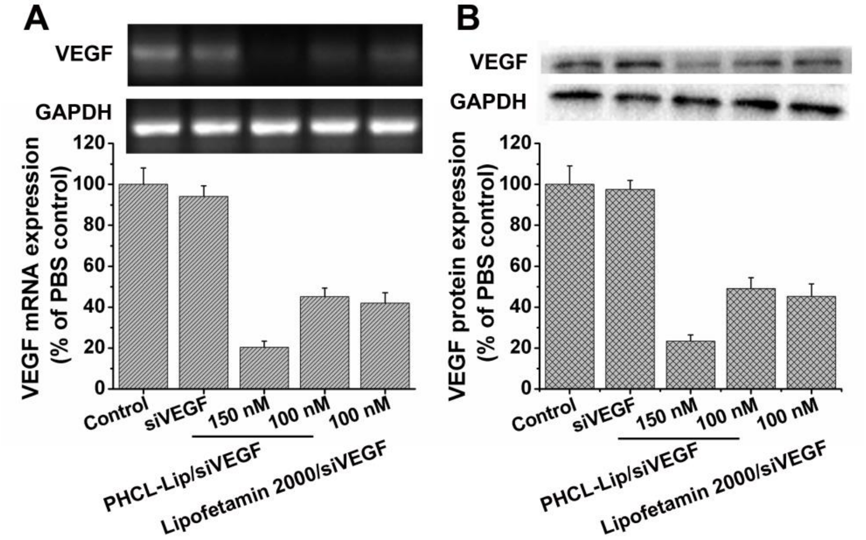

Figure S7. VEGF mRNA level (A) via RT-PCR assay and VEGF protein level via western blot analysis of A549 cells treated with PHCL-Lip/siVEGF and Lipofectamin 2000/siVEGF.

1.9 Effect of VEGF downregulation on growth of HUVEC

Briefly, A549 cells were incubated with PBS control, rare siVEGF, P-Lip/siVEGF at pH 7.4 or 6.5, PHCL-Lip/siVEGF at pH 7.4 or 6.5 for 48 h. The culture medium was taken out for further study. HUVEC cells was seeded in 96-well plates at a density of 5000 cells per well and incubated for 24 h with complete medium, followed by 48 h of hunger treatment with FBS-free medium. Subsequently, HUVEC cells were exposed to the culture medium above for 48 h and cell viability of various group were measured *via* MTT assay. The relative cell viability of siVEGF formulation-treated group to PBS –treated control group was calculated to evaluate the promoting effect of VEGF downregulation on growth of HUVEC.

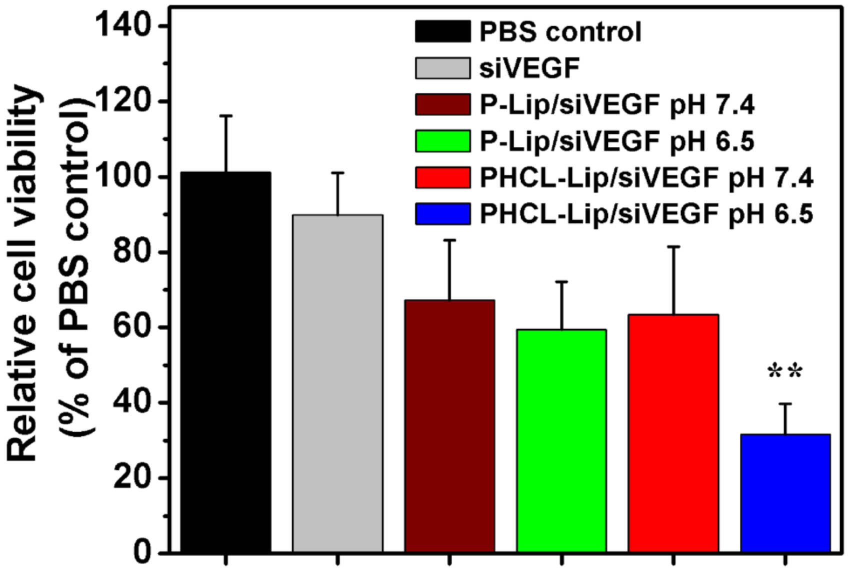

Figure S8. Relative cell viability (% of PBS control) of HUVEC treated with the culture medium of different formulations-treated A549 cells for 48 h. (n=5)

1.10 *In vitro* motility and invasion ability analysis

Wound-healing assay and matrigel invasion assay were carried out to investigate whether VEGF secretion from A549 cells after transfected with PHCL-Lip/siVEGF or P-Lip/siVEGF NPs would make influence on HUVEC migration and invasion. In wound-healing assay, A549 cells were treated with various formulations and then the condition medium was collected. HUVEC were seeded on 6-well plates with 5×10^5 cells per well. After 12 h of hungry treatment, HUVEC were incubated with the above condition medium for 24 h. Then, the cells were horizontally scratching through the well per 1 cm and washed with PBS for three times to remove the dropped cells. To

evaluate the migration ability of treated cells, the plates were imaged at 0, 24 and 48 h via a fluorescence microscope (RX51, Olympus), respectively.

The matrigel invasion assay was performed using transwell (Corning) where the upper surface was coated with FBS-free diluted matrigel (200 μg/mL). 2×10^5 of HUVEC incubated with the above condition medium were seeded on the upper chamber surface and complete culture medium was added into the lower chamber. Cells were then incubated for 0, 24 or 48 h and non-migrated cells on the upper surface of the membrane were gently removed with a cotton swab before staining the bottom surface with crystal violet staining solution. Cells were imaged *via* the fluorescence microscopy above.

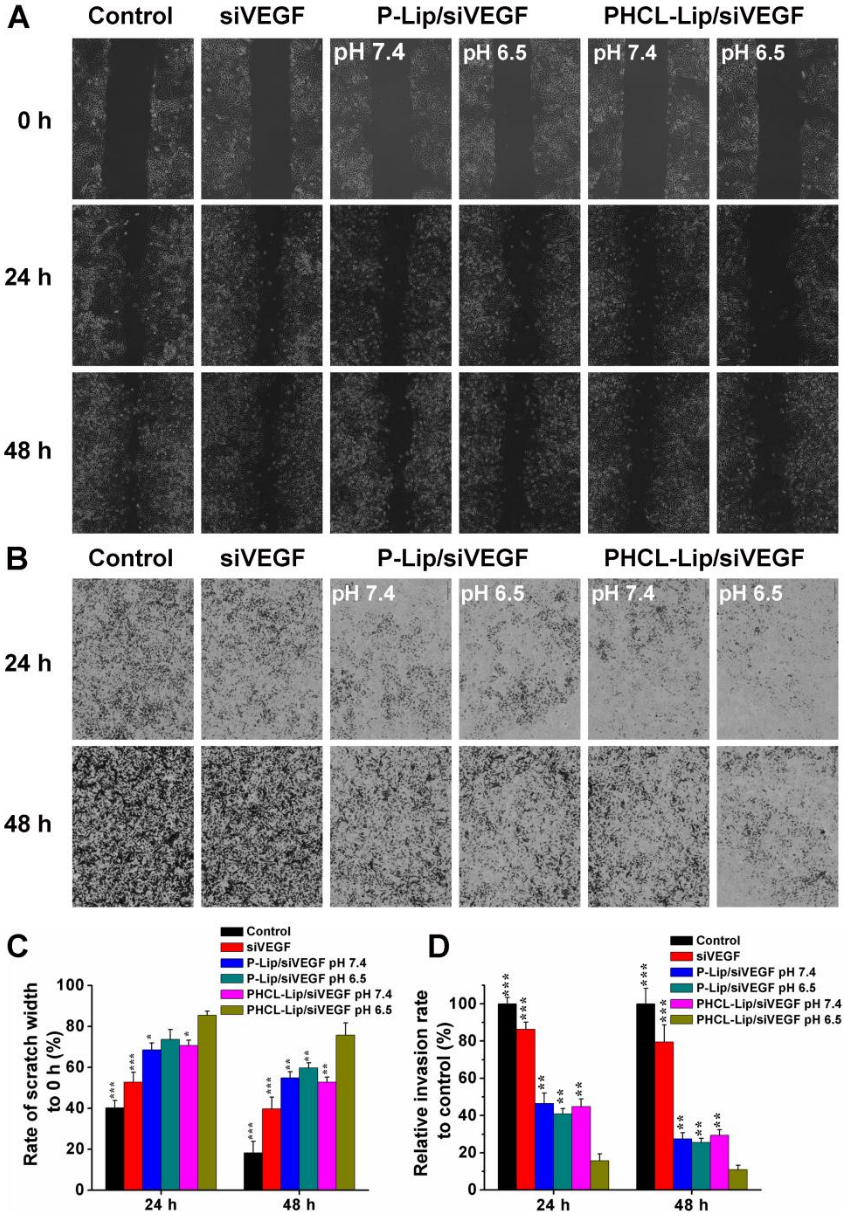

Figure S9. (A) *In vitro* migration ability via wound-healing assay and (B) invasion ability *via* matrigel invasion assay of HUVEC cells after treated with different condition medium of siVEGF formulations-treated A549 cells. (C) Relative scratch width and (D) relative invasion rate of cells at 24 h or 48 h.

As shown in Figure S9, siVEGF-encapsulated formulations showed much wider scratching width than control and rare siVEGF group at both 24 and 48 h. Significantly, cells treated with PHCL-Lip/siVEGF at pH 6.5 showed no obvious mobility to the scratches center at 24 h than that at pH 7.4. And this difference was more visible at 48 h (Figure 3E). Similarly, matrigel invasion assay revealed that PHCL-Lip/siVEGF at pH 6.5 group performed the least amount of migrated HUVEC cells at either 24 h or 48 h (Figure 3D and 3F), which might be due to the most excellent downregulation effect of VEGF expression. Non-coated P-Lip formulation exhibited no significant difference at pH 7.4 and 6.5. These results suggested that PHCL-Lip/siVEGF could significantly reduce VEGF secretion especially at tumor slightly acidic environment and consequently weaken the migration and invasion ability of endothelial cells effectively.

1.11 *In vitro* safety evaluation

In vitro safety of various blank nanoparticles was evaluated *via* MTT assay. Briefly, the cytotoxicity of blank PHCL polymer, Lip, P-Lip and PHCL-Lip against A549 cells and BEAS-2B cells was investigated.

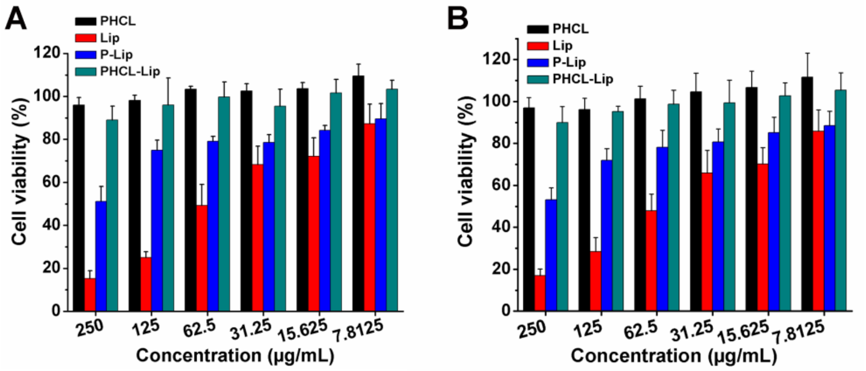

Figure S10. Cytotoxicity of blank PHCL polymer, Lip, P-Lip and PHCL-Lip against A549 cells (A) and BEAS-2B cells (B).

1.12 The accumulation of NPs in tumor lesion and normal lung tissues

Drug delivery of NPs is significant to enhance therapeutic efficacy. In this point of view, the accumulation of NPs in the tumor lesion and normal lung tissues were

observed. In brief, we established the orthotopic Luc-A549 tumor bearing nude mice models. These nude mice were randomly divided into two groups and were intravenously administrated with P-Lip/NR or PHCL-Lip/NR with NR of 13.5 mg/kg (NR referred to the fluorescence tracing dye Nile Red). 24 h or 48 h post administration, mice were sacrificed. Tumor lesion and normal lung tissues were harvested and fixed with formaldehyde (10%). The frozen slices of tumor tissues were obtained followed by staining the cell nuclei with Hoechst 33258. The accumulation of NPs in tumor lesion and normal lung tissues were then observed by a CLSM. As shown in Figure S11, both P-Lip/NR and PHCL-Lip/NR were mainly accumulated in tumor lesion in 24 h or 48 h. Besides, significantly higher accumulation of PHCL-Lip/NR in tumor lesion was observed. These results suggested that these NPs were significantly accumulated in tumor lesion rather than normal lung tissues and PHCL-Lip exhibited better tumor targeting efficacy.

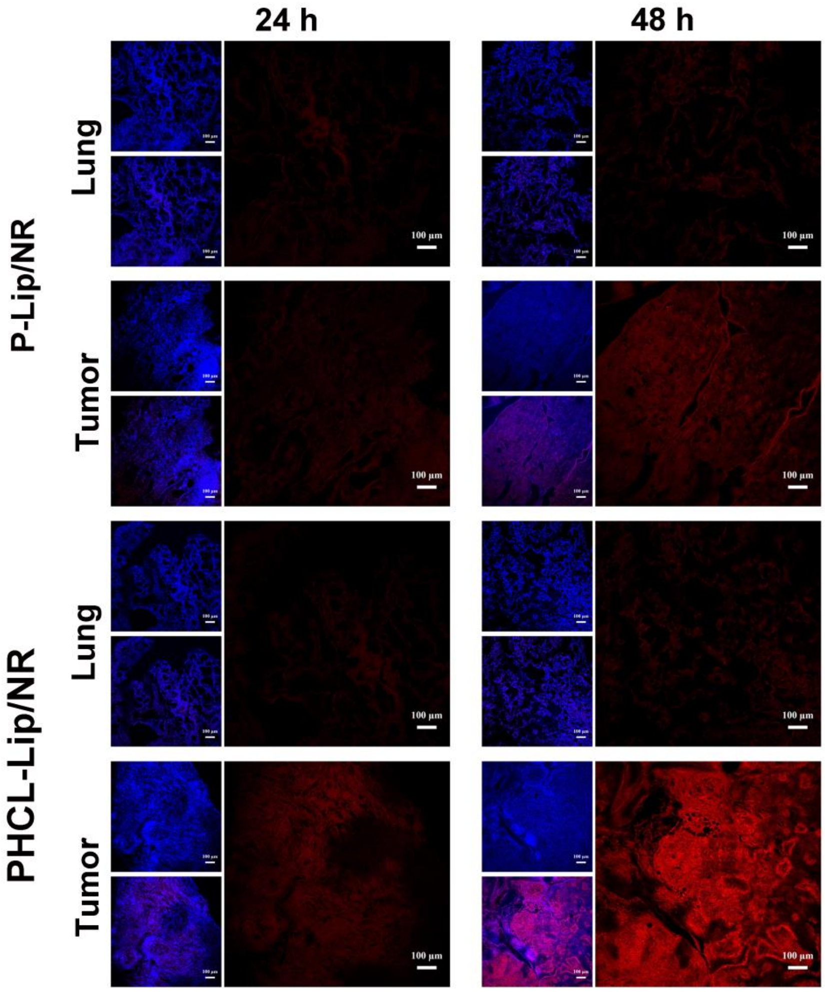

Figure S11. The fluorescence distribution of NR encapsulated in NPs in tumor lesion and normal lung tissues after intravenous administration. NR concentration was 13.5 mg/kg. Blue fluorescence represented Hoechst 33258 stained nucleus and red fluorescence represented NR.

1.13 Immunofluorescence analysis of tumor vascular

Immunofluorescence analysis was performed to observe the microvessels distribution and density in tumor tissues. In brief, the excised tumor tissues after treatment were pre-frozen at -80 °C and then cut into 10 μm-thick frozen slices, followed by fixed with acetone for 10 min. Subsequently, these frozen slices were blocked with PBS containing 5% BSA at room temperature for 1 h and subsequently incubated with anti-CD34 antibody (1:200 dilution) at room temperature for another 1 h. After washing with pre-cold 4 °C PBS, Alexa Fluor® 647-conjuageted secondary antibody (1:400 diluton) was used to bind the anti-CD44 antibody to label the tumor vessels as red. Hoechst 33258 (blue) was used as the nuclear counter staining. The prepared slices were imaged via CLSM to monitor the microvessels distribution and density in orthotopic tumor tissues to evaluate the anti-angiogenesis efficacy.

1.14 Blood chemistry test and hematological analysis

For the safety evaluation, blood chemistry test and hematological analysis of mice after treatment were conducted. In brief, after administrated with ETO and siVEGF formulations for total 5 doses, complete blood samples were collected from the abdominal aorta of mice for the complete blood panel analysis. The key hematological markers including white blood cells (WBC), red blood cells (RBC), hemoglobin (HGB), platelets (PLT) and lymphocytes (Lymph) were evaluated. As shown in Figure S12, all above markers in mice treated with Lip-encapsulated ETO formulations showed similar level with the control group. However, some decrease in the level of these parameters especially WBC level with ETO injection treatment was found.

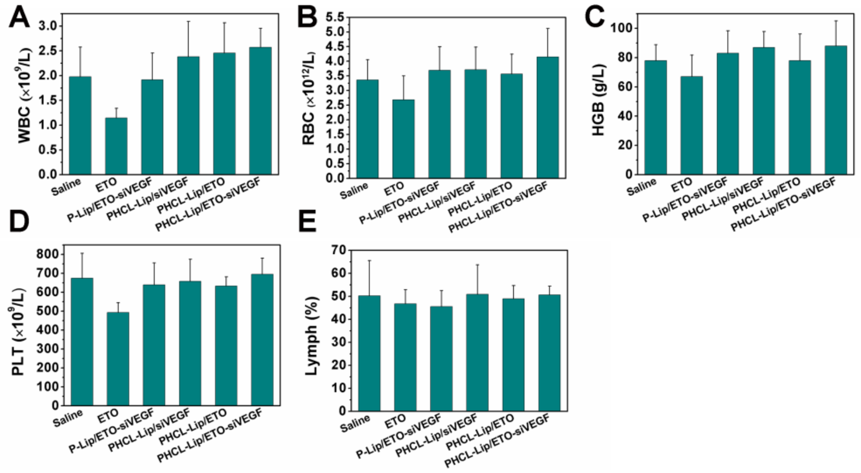

Figure S12. A complete blood count analysis after administration with different formulations

Similarly, blood samples were collected with the same treatment followed by centrifugation with 12000 rpm. The supernatant serum was collected for blood chemistry test. As shown in Figure S13, no significant difference was observed between Saline and different Lip-encapsulated ETO formulations in terms of the alanine aminotransferase (ALT), aspartate aminotransferase (AST), alkaline phosphatase (ALP), creatine kinase (CK), urea nitrogen (UREA) and creatinine (CREA) levels. However, ETO injection induced higher level of these biochemistry indexes. The results of the biochemical and hematological analysis indicated that different Lip-encapsulated ETO formulations evidently reduced the toxicity of ETO *in vivo*.

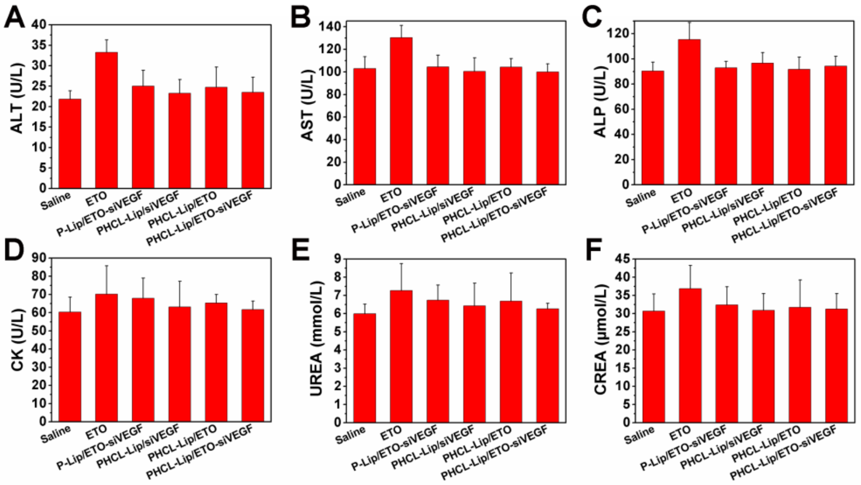

Figure S13. Blood chemistry profile analysis after administration with different formulations

1.15 *In vivo* VEGF downregulation evaluation

The expression levels of VEGF in tumor tissues after treatment with various formulations were detected via western blot assay. Briefly, the excised lung (tumor) after treatment was homogenized in PBS and centrifuged with 13000 rpm to obtain the supernatant followed by extracting VEGF protein for western blot analysis. The result was shown in Figure S14.

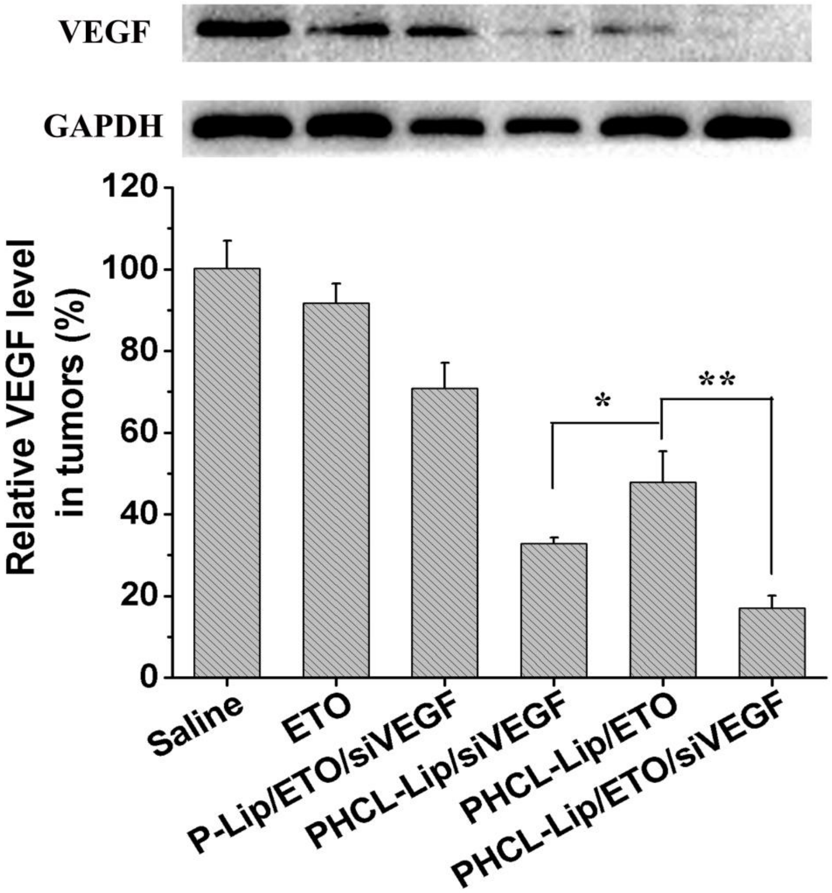

Figure S14. Relative VEGF levels in orthotopic A549 tumors after various formulations administration (n=5).

1.16 H&E analysis of spleen and kidney of mice after different treatment

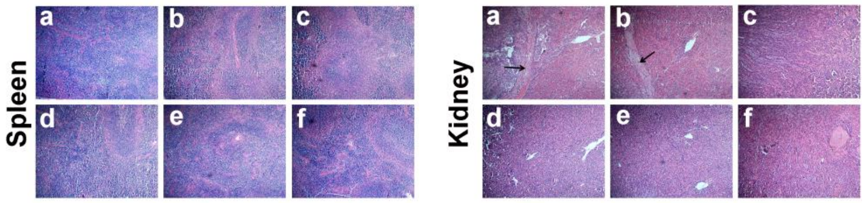

Figure S15. Representative H&E images of spleen and kidney of mice after different formulations treatment. Different formulations represented a. Saline, b. ETO injection, c. P-Lip/ETO-siVEGF, d. PHCL-Lip/siVEGF, e. PHCL-Lip/ETO, f. PHCL-Lip/ETO-siVEGF. Data was represented as mean ± SD (n=5).

1.17. Evaluation of the inflammatory response in lung tumors and the surrounding lung tissues before/after treatments

Considering anti-angiogenic therapy and chemotherapy may cause immune responses and trigger inflammation in tumor microenvironment, the levels of canonical inflammation-associated cytokines including IFN-γ, IL-6, IL-1β and TNF-α in the lung tumors and the surrounding normal lung tissues of mice before/after PHCL-Lip/ETO-siVEGF treatment were measured by ELISA. As shown in Figure S16, mice after PHCL-Lip/ETO-siVEGF treatments showed higher inflammatory response in lung tumors than those before treatments. No significant increase was found in the surrounding normal tissues of mice between before and after PHCL-Lip/ETO-siVEGF treatments. Besides, the lung tumors exhibited much higher inflammation-associated cytokines level than the surrounding normal tissues, which might be due to the immune responses induced by anti-angiogenic therapy and chemotherapy.

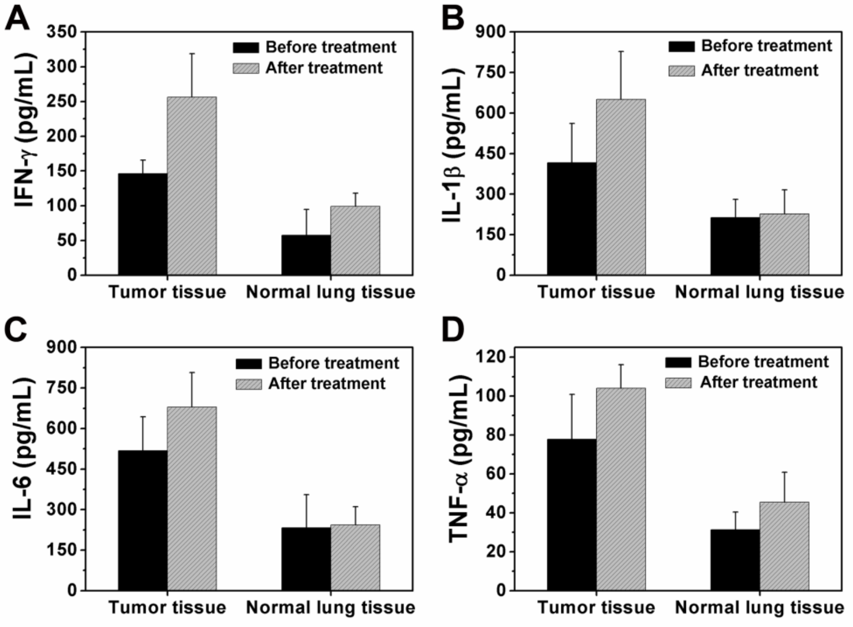

Figure S16. Inflammation-associated cytokines including IFN-γ (A), IL-1β (B), IL-6 (C), and TNF-α (D) in the lung tumors and the surrounding normal lung tissues of mice before/after PHCL-Lip/ETO-siVEGF treatment were measured by ELISA (n=5). Data was represented as mean ± SD.

1.18. The survival rate of mice treated with various formulations

The survival rate of mice treated with various formulations was evaluated. Briefly, nude mice bearing orthotopic Luc-A549 tumor model were established and administrated intravenously with various formulations 14 days after the inoculation. Mice received a total of 5 doses with each dose given every 3 days. At the end of the observation period, survival curve were obtained by the Kaplan-Meier analysis (n=5). “Time (d)” represented days from the start of treatment. As shown in Figure S17, the enhanced inhibition of tumor proliferation and metastasis by the combined therapy with PHCL-Lip/ETO-siVEGF achieved prolonged survival than other treatments.

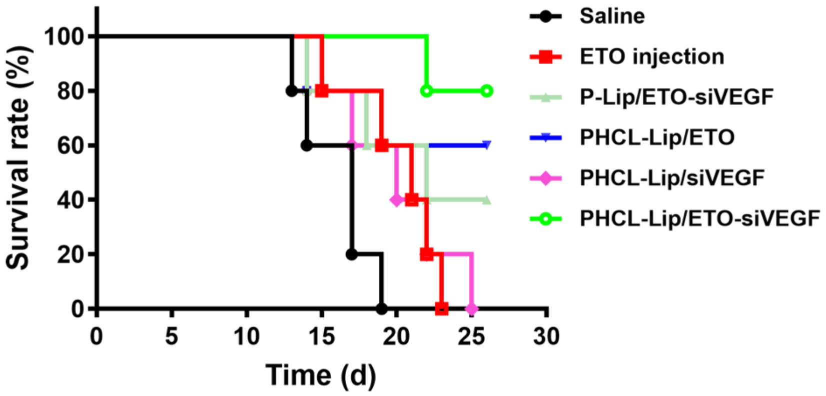

Figure S17. Survival rate of mice after treatment with various formulations; “Time (d)” represented days from the start of treatment (n=5).

References:

1. Li F, Chen WL, You BG, Liu Y, Yang SD, Zhang XN, et al. Enhanced cellular internalization and on-demand intracellular release of doxorubicin by stepwise ph-/Reduction-Responsive Nanoparticles. ACS Appl Mater Interfaces. 2016; 8: 32146-58.
2. Yuan YY, Mao CQ, Du XJ, Du JZ, Wang F, Wang J. Surface charge switchable nanoparticles based on zwitterionic polymer for enhanced drug delivery to tumor. Adv Mater. 2012; 24: 5476-80.
3. Patil S, Sandberg A, Heckert E, Self W, Seal S. Protein adsorption and cellular uptake of cerium oxide nanoparticles as a function of zeta potential. Biomaterials. 2007; 28: 4600-7.

2. Abbreviations

NSCLC: non-small-cell lung cancer;

ETO: etoposide;

GSH: glutathione;

EPR: enhanced permeability and retention;

PHCL: PEGylated histidine-grafted chitosan-lipoic acid;

VEGF: vascular endothelial growth factor;

DOTAP: 1,2-Dioleoyl-3-trimethylammonium-propane chloride salt;

P-Lip: PEGylated cationic liposomes;

PHCL-Lip: PHCL coated cationic liposomes;

TEM: transmission electron microscope;

NR: nile red;

CLSM: confocal laser scanning microscope;

$siRNA^{FAM}$: FAM labeled siRNA;

FACS: fluorescence activated Cell Sorter;

RT-PCR: reverse transcription PCR;

HPLC: high performance liquid chromatography;

MWCO: molecular weight cut-off.

3. Supplemental Tables

Table S1. Characterization of PHCL-Lip NPs and P-Lip NPs

Group	Size (nm)	Zeta potential (mV)	EE (%)	DL (%)
PHCL-Lip	176.5 ± 6.3	-12.06 ± 1.85	89.88 ± 1.52	15.24 ± 1.07
P-Lip	148.7 ± 3.2	26.61 ± 0.92	91.25 ± 2.37	17.19 ± 1.82
